# The enmity paradox

**DOI:** 10.1038/s41598-023-47167-9

**Published:** 2023-11-16

**Authors:** Amir Ghasemian, Nicholas A. Christakis

**Affiliations:** Yale Institute for Network Science, Yale University, New Haven, CT 06511 USA

**Keywords:** Computational science, Applied mathematics

## Abstract

The “friendship paradox” of social networks states that, on average, “your friends have more friends than you do”. Here, we theoretically and empirically explore a related and overlooked paradox we refer to as the “enmity paradox”. We use empirical data from 24,678 people living in 176 villages in rural Honduras. We empirically show that, for a real negative undirected network (created by symmetrizing antagonistic interactions), the paradox exists as it does in the positive world. Specifically, a person’s enemies have more enemies, on average, than a person does. Furthermore, in a mixed world of positive and negative ties, we study the conditions for the existence of the paradox, which we refer to as the “mixed-world paradox”, both theoretically and empirically, finding that, for instance, a person’s friends typically have more enemies than a person does. We also confirm the “generalized” enmity paradox for non-topological attributes in real data, analogous to the generalized friendship paradox (e.g., the claim that a person’s enemies are richer, on average, than a person is). As a consequence, the naturally occurring variance in the degree distribution of both friendship and antagonism in social networks can skew people’s perceptions of the social world.

## Introduction

The empirical observation that a person’s friends have, on average, more friends than they do is called the “friendship paradox”^1^. The friendship paradox can be explained by the fact that, in computing the average degree of individuals’ friends, high-degree individuals are counted more than individuals with low degree. It is a kind of sampling bias. Computing the average degree based on a person’s network neighbors’ perspective is biased towards a higher mean value than computing it from a person’s own perspective.

The friendship paradox has been generalized to non-topological characteristics in social networks, such as happiness and wealth (people’s friends are happier and richer than they are, on average)^2^. The positive correlation between network degree and various characteristics is the origin of this generalization. The friendship paradox has also been used in social network polling and estimating power-law degree distributions^3^, and it has been studied in relation to some other topological properties of networks, such as betweenness, closeness, eigenvector, and Katz centrality^4,5^, as well as extensions to directed networks^5,6^. Moreover, the friendship paradox can be considered a special case of human social sensing^7^. Furthermore, individuals with more social connections are more indicative of early trends than the average member of the population for many societal phenomena, from the spread of disease^8^ to the spread of information^9^. It is even possible to exploit the friendship paradox to develop effective strategies to intervene in networks^10–13^.

Recent work has examined what topological features influence the “strength” of friendship paradox. The magnitude of difference between the average of a random node’s degree and the average of that node’s neighbors’ degrees is the “local” strength of the friendship paradox. In contrast, the magnitude of difference between the average degree of a random node and the average degree of a random neighbor of a random node is the “global” strength. For the so-called “local” formulation of the friendship paradox, for three classes of network models (the Poisson random graph, the configuration model, and a model of a random degree-assortative network), it has been shown that networks with more heterogeneous degree distributions and with negative assortativity tend to have the strongest friendship paradox^14^. Two formulations of the friendship paradox have been examined based on the “global” and “local” structure of a network^1^. By exploiting a topological property called “inversity,” i.e., the Pearson correlation between a node’s degree and the inverse degree of its neighbors, it is possible to evaluate the relationship between these two formulations and the strength of the friendship paradox^12,15^. Similar discussions for the generalized friendship paradox in directed networks are possible^6^.

To date, the friendship paradox has been investigated primarily from the perspective of positive networks, and little attention has been paid to questions regarding a world in which solely negative ties exist or a world in which both positive and negative ties exist. As a result, it is unclear whether, empirically speaking, we can observe these paradoxes with respect to antagonistic ties. Does a negative world manifest the same paradoxes? If so, what mechanisms would cause such paradoxes to occur in a negative world?

Although there is an inherent mathematical symmetry between positive and negative network objects, which theoretically supports the existence of the enmity paradox, it is uncertain if this paradox exists empirically. Since the positive and negative worlds are empirically distinct, the *strength* of the paradox is unclear. As an example, the variance in degree, the primary factor affecting the strength of these paradoxes, might be much smaller in the negative world (as we will show), with consequent implications. Thus, here, we explore the “enmity paradox”, by which we mean, most generally, that the mean number of enemies a person has is *lower* than the mean number of enemies their enemies have. Furthermore, we examine whether this paradox is observed in a mixed world of both positive and negative ties by comparing a person’s average number of friends with their enemies’ average number of friends, and by comparing a person’s average number of enemies with their friends’ average number of enemies. In this case, we refer to the paradox as the “mixed-world paradox”, and we propose some theoretical innovations as well.

## The enmity paradox

It is a fact of mathematics that, in a population with variance in the degree distribution (and subject to certain provisos), the friendship paradox exists. But its extent is very much a result of underlying social factors (such as variation across people in the number of friends they want, or whether popular people are preferred as friends). Some aspects of this phenomenon can also be self-reinforcing as a consequence of positive feedback that results from the biased perception^16^.

However, even though there is an intrinsic mathematical symmetry between positive and negative network objects (as instantiated by adjacency matrices), which theoretically supports the existence of the enmity paradox, the empirical existence and *strength* of the paradox are not assured. We mathematically clarify and then empirically investigate the existence, origins, and manifestations of the enmity paradox.

First, we review the equations for the enmity paradox, which are similar to the equations derived for the friendship paradox^1^. Based on our comparison of the mean number of enemies of individuals with the mean number of enemies’ enemies in the negative world, we theoretically assert that the enmity paradox should indeed arise in a similar manner to the friendship paradox.

Consider a simple signed network $G=(V, E_{(+)}, E_{(-)})$, where *V* is the vertex set with *n* nodes, and $E_{(+)}$ and $E_{(-)}$ are the set of positive and negative edges revealing two not-necessarily-dependent worlds. The adjacency matrices corresponding to the positive and negative interactions are denoted as $A_{(+)}$ and $A_{(-)}$, respectively. In order to create undirected networks, we either (1) remove unreciprocated edges from the network, or (2) symmetrize the network by removing the edges’ direction. For example, the node *j* is considered a neighbor of the node *i* if, in the former, both edges (*i*, *j*) and (*j*, *i*) are included in the set *E*, while, in the latter, if at least one of these edges exist in the edge set. An edge is referred to as a “friend” if it belongs to $E_{(+)}$, and as an “enemy” if it belongs to $E_{(-)}$. In addition, we also refer to a node *j* as an $\ell$-hop neighbor of node *i* if there is a walk with length $\ell$ between *i* and *j*; a walk can include repeated nodes. Here, we focus solely on the enmity and friendship paradoxes for undirected networks. The enmity paradox for directed networks is provided in the Supplementary Material, Sect. D.

There are two types of degrees for each node *i* in these two parallel worlds, $k_{(+),i} = \sum _j A_{(+),ij}$ corresponding to positive network, and $k_{(-),i}= \sum _j A_{(-),ij}$ corresponding to negative network. The probability of a node with degree $k_{(+)}$ ($k_{(-)}$) is denoted as $p_{k_{(+)}}^{(0)}$ ($p_{k_{(-)}}^{(0)}$) or, more simply, $p_{k_{(+)}}$ ($p_{k_{(-)}}$); and the probability of a node’s friend with positive degree $k_{(+)}$ and negative degree $k_{(-)}$ is denoted as $p_{k_{(+)}}^{(1)}$ and $p_{k_{(-)}}^{(1)}$, respectively. Similarly, the probability of a node’s enemy with positive degree $k_{(+)}$ and negative degree $k_{(-)}$ is denoted as $q_{k_{(+)}}^{(1)}$ and $q_{k_{(-)}}^{(1)}$, respectively. For simplicity, whenever it is clear from context, we denote the degree, the degree distribution, and the degree distributions of neighboring nodes corresponding to the friendship and enmity networks using *k*, $p_{k}$, and $p_{k}^{(1)}$ and $q_{k}^{(1)}$.

To establish the enmity paradox, we need to compare the average negative degree of a random node with the average negative degree of a random enemy of a random node. The average negative degree of a random node can be written as $\langle k_{(-)} \rangle _{p_{k_{(-)}}} = \sum _{k_{(-)}} k_{(-)} p_{k_{(-)}}$. Going along a random negative edge to one of its enemies leads to a node with negative degree $k_{(-)}$ with probability $q_{k_{(-)}}^{(1)}$ proportional to $k_{(-)} p_{k_{(-)}}$, i.e., $q_{k_{(-)}}^{(1)} = k_{(-)} p_{k_{(-)}}/\langle k_{(-)} \rangle _{p_{k_{(-)}}}$. Therefore, the average degree of a random enemy can be written as $\langle k_{(-)} \rangle _{q_{k_{(-)}}^{(1)}} = \sum _{k_{(-)}} k_{(-)} q_{k_{(-)}}^{(1)} = \sum _{k_{(-)}} {k_{(-)}}^2 p_{k_{(-)}}/\langle k_{(-)} \rangle _{p_{k_{(-)}}}$ for enmity networks. Using Jenson’s inequality, it can be shown that $\langle k_{(-)} \rangle _{q_{k_{(-)}}^{(1)}} \ge \langle k_{(-)} \rangle _{p_{k_{(-)}}}$. This inequality can be called the “enmity paradox” for the negative world, and it follows from the same mathematical facts as the friendship paradox in the positive world (Table 1).

**Table 1:** The glossary of paradoxes.

Paradox	Description
Friendship paradox	On average, your friends have more friends than you do
Enmity paradox	On average, your enemies have more enemies than you do
Mixed-world paradox	On average, your friends/enemies have more enemies/friends than you do

Social networks that simultaneously involve both positive and negative ties are theoretically more complicated. That is, analytically, we have shown that people have fewer enemies on average than their enemies; however, the result of mixing positive and negative worlds is not obvious. Do people have more or fewer enemies than their friends, or do they have more or fewer friends than their enemies? To address these questions, we derive equations for the mixed-world paradox (Table 1).

Considering a dependency between positive and negative degrees with the correlation denoted by $\rho _{k_{(+)},k_{(-)}} =\left( E\left[ {k_{(+)}k_{(-)}}\right] -E{k_{(+)}}E{k_{(-)}}\right) /\sigma _{k_{(+)}}\sigma _{k_{(-)}}$, we have the joint probability of degrees $k_{(+)}$ and $k_{(-)}$ as $p_{k_{(+)},k_{(-)}} = f(\sigma _{k_{(+)},k_{(-)}})$. For this scenario, the average degree of a person’s enemies would be denoted by $\langle k_{(-)}\rangle _{p_{k_{(-)}}}$; the average degree of a person’s friends would be denoted by $\langle k_{(+)}\rangle _{p_{k_{(+)}}}$; the average degree of a person’s friends’ enemies would be denoted by $\langle k_{(-)} \rangle _{p_{k_{(+)},k_{(-)}}^{(1)}}$; and the average degree of a person’s enemies’ friends would be denoted by $\langle k_{(+)} \rangle _{q_{k_{(+)},k_{(-)}}^{(1)}}$, where the first two are driven before and the last two are as follows.

1a $$\begin{aligned} \langle {k_{(-)}} \rangle _{p_{k_{(+)},k_{(-)}}^{(1)}}&= \sum _{k_{(+)}, k_{(-)}} k_{(-)}k_{(+)} p_{k_{(+)},k_{(-)}}\big /\langle k_{(+)}\rangle _{p_{k_{(+)}}}, \end{aligned}$$

1b $$\begin{aligned} \langle {k_{(+)}} \rangle _{q_{k_{(+)},k_{(-)}}^{(1)}}&= \sum _{k_{(+)}, k_{(-)}} k_{(+)}k_{(-)} p_{k_{(+)},k_{(-)}}\big /\langle k_{(-)}\rangle _{p_{k_{(-)}}}. \end{aligned}$$

Therefore, we have three different regimes as follows: (1) If we have independent positive and negative worlds, i.e., $p_{k_{(+)},k_{(-)}} = p_{k_{(+)}}p_{k_{(-)}}$, then $\langle k_{(-)} \rangle _{p_{k_{(+)},k_{(-)}}^{(1)}} = \langle k_{(-)}\rangle _{p_{k_{(-)}}}$ and there is no difference between the average number of a person’s enemies and average number of enemies of a person’s friends, and similarly $\langle k_{(+)} \rangle _{q_{k_{(+)},k_{(-)}}^{(1)}} = \langle k_{(+)}\rangle _{p_{k_{(+)}}}$, which means no difference between the average number of a person’s friends and the average number of a person’s enemies’ friends. (2) If there is a positive correlation between the two worlds, i.e., $\rho _{k_{(+)},k_{(-)}} > 0$ (or when $\sigma _{k_{(+)},k_{(-)}} > 0$), then we have paradoxes in the mixed world in the same direction as the enmity and friendship paradoxes, as a person’s friends have more enemies and a person’s enemies have more friends compared to a person. (3) Finally, if there is a negative correlation between the two worlds, i.e., $\rho _{k_{(+)},k_{(-)}} < 0$ (or when $\sigma _{k_{(+)},k_{(-)}} <0$), then we have paradoxes in the mixed world in the opposite direction with the enmity and friendship paradoxes, as a person’s friends have fewer enemies and a person’s enemies have fewer friends compared to the person.

### Computation and representation of enmity paradox for empirical data

**Figure 1:**
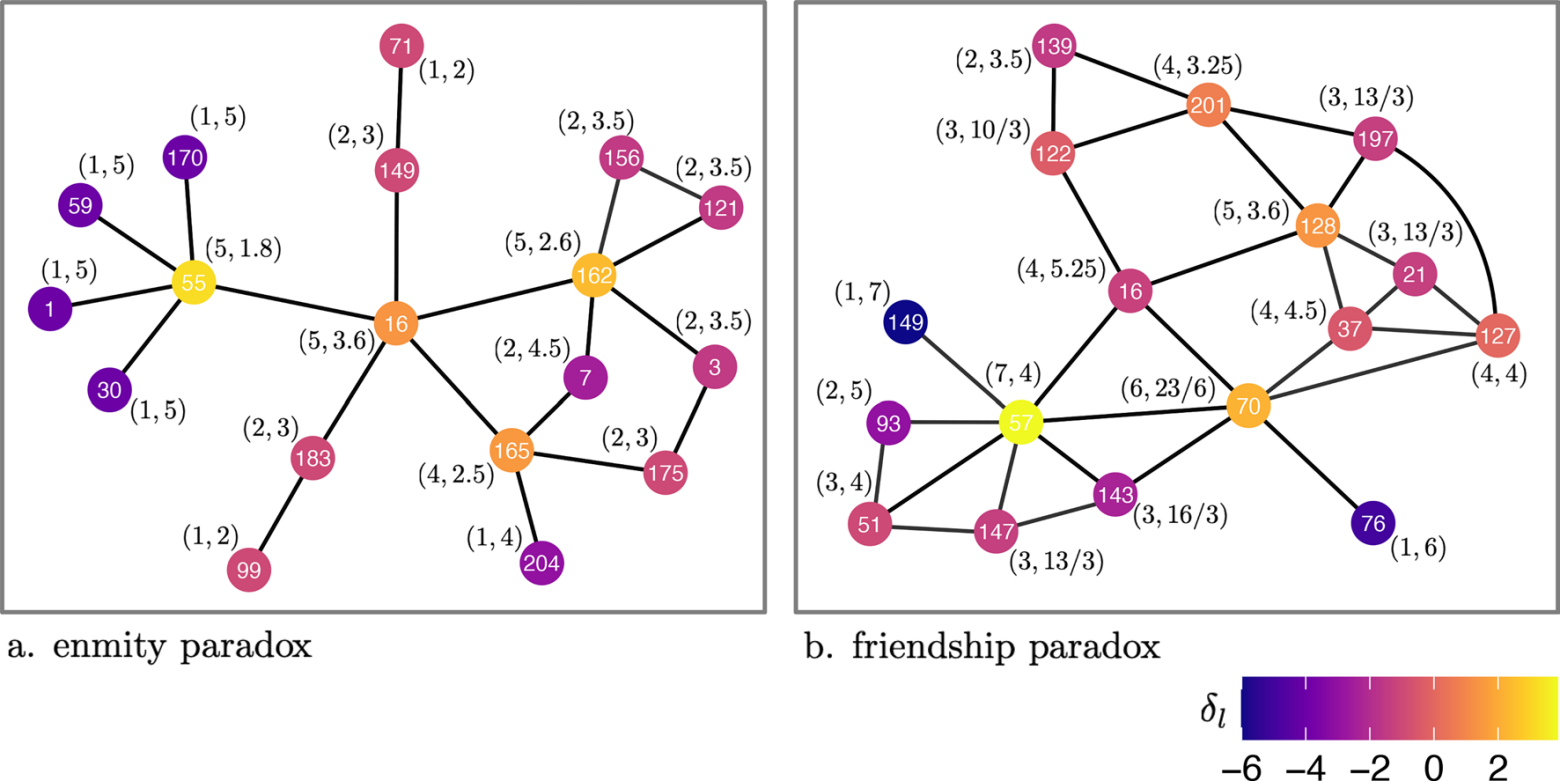
Global versus local enmity and friendship paradoxes. The networks are induced graphs for vertices at distance 2 from one vertex in one village in Honduras. On the left is the enmity network constructed from enmity interactions, and on the right is the friendship network constructed from friendship interactions. Darker blue represents nodes with maximum paradox, while red represents nodes with balance, and yellow represents nodes with the opposite circumstance. The (*a*, *b*) value for each node represents the (mean degree, mean neighbors’ degree) and the difference, i.e., $a-b$, represents the individual local paradox. It is the mean of all individual local paradoxes that constitutes the “local paradox.” In the case of the enmity paradox, the local paradox is equal to $- \,1.25$, while in the case of the friendship paradox, it is equal to $- \,1.04$. The “global paradox” is the difference between the mean degree of a random node and the mean degree of a random endpoint on a random edge. Accordingly, the global enmity paradox is equal to $-\, 0.93$ and the global friendship paradox is equal to $- \,0.69$ here.

Investigating the enmity and friendship paradoxes in practice requires writing the equations using the data—as we are given neither the generative probability of the ties nor the joint probability of negative and positive ties. The friendship paradox has previously been formulated in two different variations which relate to global bias and local bias^1,6^. In the “global” formulation, we compare the average degree of a random node *i* with the average degree of a random neighbor of a random node *j*—or a random end of a random edge. In the “local” formulation, we compare the difference between the average of a random node’s degree and the average of that node’s neighbors’ degrees locally (Fig. 1). From now on, by “paradox strength”, we mean the magnitude of the global difference $\delta _g$ or of local difference $\delta _l$, as specified.

The paradox in the global variation is the result of the oversampling of high-degree nodes. In the local variation, the paradox can be intensified locally if there is a positive correlation between a node’s degree and the inverse degree of its neighbors, or it can be attenuated if there is a negative correlation between these variables. This correlation measure is known as “inversity”, and, although it is related to degree assortativity, it is not the same^12^; the relevance of such metrics has also been previously explored empirically^15^.

The enmity paradox can similarly be defined for local and global formulations. We first redo the empirical computations for the enmity and friendship paradoxes using empirical data, followed by a matrix representation of the enmity and friendship paradoxes. These equations (Eqs. 2–5) present a novel matrix algebraic derivation of the enmity paradox in the negative world that may also (unsurprisingly) be applied to the friendship paradox due to the symmetry in representing positive and negative objects.

The global formulation of the enmity paradox is the comparison between the average degree of a random node *i*, i.e., $\sum _i k_i/n$ and the average degree of its neighbors’ degrees, i.e., $\sum _i k_i^2/\sum _i k_i$, where the difference can be written using matrix formulation as Eq. (2),

2 $$\begin{aligned} \delta _{g,-w}(-) =&\frac{({\textbf{1}}^TA_{(-)}{\textbf{1}})^2 - {\textbf{1}}^TA_{(-)}^2 {\textbf{1}}\cdot {\textbf{1}}^T{\textbf{1}}}{{\textbf{1}}^TA_{(-)}{\textbf{1}}\cdot {\textbf{1}}^T{\textbf{1}}}, \end{aligned}$$

where ${\textbf{1}}$ is a vector of ones with length *n* (${\textbf{1}}^T{\textbf{1}}=n$). In this notation, the $-w$ and $+w$ indicate the type of one’s neighbor, as one’s enemy and friend, respectively. The $(-)$ and $(+)$ denote the type of comparison as enemies or friends. This global quantity is a non-positive value due to the Cauchy–Schwarz inequality ($|\langle \textbf{u},\textbf{v} \rangle |^2\le \langle \textbf{u}, \textbf{u} \rangle \langle \textbf{v}, \textbf{v} \rangle$) with $\textbf{u} = A{\textbf{1}}$ and $\textbf{v} = {\textbf{1}}$, where the equality happens when $\textbf{u} = k \textbf{v}$, i.e., when we have a *k*-regular network. As a result, degree heterogeneity is directly related to the enmity “paradox strength,” since the numerator of the right-hand side in Eq. (2) is the negative variance of the negative degree distribution. These results are similar to the results for the friendship paradox.

For the local formulation, we have the difference between each individual’s degree and the average degree of its neighbors, which is reminiscent of a Laplacian matrix acting on the degree vector *d*, where *i*-th outcome of this operation can be written as $\Delta _i = k_i - \sum _j A_{ij} k_j/k_i$; and, by averaging out these outcomes, we have the local formulation of enmity paradox as Eq. (3),

3 $$\begin{aligned} \delta _{l,-w}(-)&=\frac{{\textbf{1}}^TA_{(-)}{\textbf{1}}- {\textbf{1}}^TD_{(-)}^{-1} A_{(-)}D_{(-)}{\textbf{1}}}{{\textbf{1}}^T{\textbf{1}}} \nonumber \\&=\frac{{\textbf{1}}^TA_{(-)}{\textbf{1}}- {\textbf{1}}^TD_{(-)} A_{(-)}D_{(-)}^{-1}{\textbf{1}}}{{\textbf{1}}^T{\textbf{1}}}, \end{aligned}$$

where $D_{(-)}$ is the diagonal matrix of negative degrees. Eq. (3) can also be written as $\delta _{l,-w}(-) ={\textbf{1}}^T L_{(-)} k_{(-)}/{\textbf{1}}^T{\textbf{1}}$, where, $L_{(-)} ={\mathbb {I}}- D_{(-)}^{-1} A_{(-)}$ is the Laplacian matrix of the negative world acting on the negative degree vector, $k_{(-)} =D_{(-)}{\textbf{1}}$. Our derivation relies on the fact that *A* is symmetric since we only consider undirected networks here (the formulations for directed networks are in the Supplementary Material, Sect. D). We can also demonstrate that this quantity is always non-positive. Since, for any two matrices *A* and *B*, we can write ${{\,\text{Tr}\,}}(AB) = {{\,\text{Tr}\,}}(BA)$, thus Eq. (3) can be written as follows,

4 $$\begin{aligned} \delta _{l,-w}(-)= \frac{{{\,\text{Tr}\,}}{\left[ A_{(-)}(2{\mathbb {J}}- D_{(-)} {\mathbb {J}}D_{(-)}^{-1} - D_{(-)}^{-1}{\mathbb {J}}D_{(-)})\right] }}{2{\textbf{1}}^T{\textbf{1}}}, \end{aligned}$$

where ${\mathbb {J}}$ is the matrix of all ones, and, due to the non-positive entries of the matrix $2{\mathbb {J}}- D_{(-)}{\mathbb {J}}D_{(-)}^{-1} - D_{(-)}^{-1}{\mathbb {J}}D_{(-)}$ for each pair (*i*, *j*) (see Ref.^12^), the resultant trace should also be non-positive.

The difference between $\delta _{g,-w}(-)$ and $\delta _{l,-w}(-)$ can be written as Eq. (5), where $\sigma _{D,(-)}^2$ is the variance of the negative degree of an endpoint of a random edge; $\sigma _{ID,(-)}^2$ is the variance of the inverse negative degree of an endpoint of a random edge; and $\overline{ k_{(-)}}$ is the average negative degree. This quantity is negative ($\delta _{g,-w}(-) < \delta _{l,-w}(-)$) if the correlation between the degree of one endpoint *i* and the inverse degree of another endpoint *j* on a random edge $(i,j)\in E_{(-)}$, i.e., $\rho _{(-)} = \text{cor}(k_{(-),i},1/k_{(-),j}|(i,j)\in E_{(-)})$ is negative; it is positive ($\delta _{g,-w}(-) > \delta _{l,-w}(-)$) if the correlation is positive; and it is zero if the correlation is zero^12^.

5 $$\begin{aligned} \delta _{g,-w}(-) - \delta _{l,-w}(-)&= \frac{{\textbf{1}}^TD_{(-)}^{-1}A_{(-)}D_{(-)}{\textbf{1}}}{{\textbf{1}}^T{\textbf{1}}} - \frac{{\textbf{1}}^TA_{(-)}^2{\textbf{1}}}{{\textbf{1}}^TA_{(-)}{\textbf{1}}} \nonumber \\&=\rho _{(-)}\overline{ k_{(-)}}\sigma _{D,(-)}\sigma _{ID,(-)}. \end{aligned}$$

For a mixed world of positive and negative edges, the equations of the mixed-world paradox can be written as follows. The global formulation of the difference between number of friends of a random node with the number of friends of enemies of a random node can be formulated as Eq. (6a). And the global formulation of the difference between the number of enemies of a random node with the number of enemies of friends of a random node can be formulated as Eq. (6b).

6a $$\begin{aligned} \delta _{g,-w}(+)&= \frac{{\textbf{1}}^TA_{(+)}{\textbf{1}}\cdot {\textbf{1}}^TA_{(-)}{\textbf{1}}- {\textbf{1}}^TA_{(+)}A_{(-)}{\textbf{1}}\cdot {\textbf{1}}^T{\textbf{1}}}{{\textbf{1}}^TA_{(-)}{\textbf{1}}\cdot {\textbf{1}}^T{\textbf{1}}}, \end{aligned}$$

6b $$\begin{aligned} \delta _{g,+w}(-)&= \frac{{\textbf{1}}^TA_{(+)}{\textbf{1}}\cdot {\textbf{1}}^TA_{(-)} {\textbf{1}}- {\textbf{1}}^TA_{(+)}A_{(-)}{\textbf{1}}\cdot {\textbf{1}}^T{\textbf{1}}}{{\textbf{1}}^TA_{(+)} {\textbf{1}}\cdot {\textbf{1}}^T{\textbf{1}}}. \end{aligned}$$

The numerator of these equations can be reduced to $\sum _i k_{(-),i} \sum _i k_{(+),i} - n\sum _i k_{(-),i}k_{(+),i}$ which is negative when the correlation between the positive and negative degrees is positive.

For the local formulation, these equations reduce to Eq. ().

7a $$\begin{aligned} \delta _{l,-w}(+)&= \frac{{\textbf{1}}^T A_{(+)}{\textbf{1}}- {\textbf{1}}^T D_{(-)}^{-1} A_{(-)} D_{(+)}{\textbf{1}}}{{\textbf{1}}^T{\textbf{1}}}, \end{aligned}$$

7b $$\begin{aligned} \delta _{l,+w}(-) =&\frac{{\textbf{1}}^T A_{(-)}{\textbf{1}}-{\textbf{1}}^T D_{(+)}^{-1} A_{(+)} D_{(-)}{\textbf{1}}}{{\textbf{1}}^T{\textbf{1}}}. \end{aligned}$$

Equation (7a) can be also written as the operation of the Laplacian matrix of the negative world on the positive degree vector $k_{(+)}$, i.e., $\delta _l = {\textbf{1}}^T L_{(-)}k_{(+)}$, where, $L_{(-)} = {\mathbb {I}}- D_{(-)}^{-1} A_{(-)}$. Similar statements can be shown regarding the relationships between $\delta _l$ and $\delta _g$ in a mixed world. The difference between $\delta _{g,-w}(+)$ (6a)/$\delta _{g,+w}(-)$ (6b) and $\delta _{l,-w}(+)$ (7a)/$\delta _{l,+w}(-)$ (7b) can be written by introducing the “generailized inversity” measure in the mixed world, i.e., the correlation between the positive degree/negative degree of one endpoint *i* and the inverse negative/positive degree of another endpoint *j* on a random negative/positive edge $(i,j)\in E_{(-)}/E_{(+)}$ (see Eqs. S34, S37 in Supplementary Material, Sect. G).

Finally, it is possible to use a similar representation to explore when the enmity paradox can be generalized for *non-topological attributes* such as happiness, wealth, and health (e.g., on average, a person’s enemies are richer or happier than they are, as has previously been shown for friendship paradox^2^). Due to the similarity between the enmity paradox and the friendship paradox, we expect that the condition for generalization of the global definition is similar to the condition previously studied for the generalized friendship paradox^2^. Similarly, the condition for the relationship between the local and global formulations of the generalized enmity paradox should be similar to the condition of their relationships for the generalized friendship paradox^6^. Derivations of these effects, and results, are provided in the Supplementary Material, Sect. F.

## Higher-order enmity paradox

We can also consider the paradoxes for higher-order neighbors. For example, in the global formulation, if the average degree of a random neighbor of a random node is greater than the average degree of a random node, is it also the case that the average degree of a random 2-hop neighbor of a random node is also larger—or for any random $\ell$-hop neighbor of a random node (an $\ell$-hop neighbor is a neighbor we arrive through a random walk with length $\ell$).

In this case, it is more specific to ask if there is a relationship between the paradox strength and the order of the neighbors. Here, the equation for global formulation of $\delta _g$ for an order of $\ell$ can be written as Eq. (8),

8 $$\begin{aligned} \delta _g^{(\ell )} =&\frac{{\textbf{1}}^TA{\textbf{1}}\cdot {\textbf{1}}^TA^{\ell }{\textbf{1}}- {\textbf{1}}^TA^{\ell +1}{\textbf{1}}\cdot {\textbf{1}}^T{\textbf{1}}}{{\textbf{1}}^TA^\ell {\textbf{1}}\cdot {\textbf{1}}^T{\textbf{1}}}, \end{aligned}$$

where for $\ell =2$ can be written as follows.

9 $$\begin{aligned} \delta _g^{(\ell =2)}&= \frac{{\textbf{1}}^TA{\textbf{1}}\cdot {\textbf{1}}^TD^{2}{\textbf{1}}- {\textbf{1}}^TDAD{\textbf{1}}\cdot {\textbf{1}}^T{\textbf{1}}}{{\textbf{1}}^TA^2 {\textbf{1}}\cdot {\textbf{1}}^T{\textbf{1}}}\nonumber \\&=\frac{{{\,\text{Tr}\,}}{\left[ A\left( {\mathbb {J}}D^2 {\mathbb {J}}- n D{\mathbb {J}}D\right) \right] }}{{\textbf{1}}^TA^2{\textbf{1}}\cdot {\textbf{1}}^T{\textbf{1}}}. \end{aligned}$$

Accordingly, we can see different scenarios under different conditions. If the degree of nodes that are connected have a positive correlation, i.e., $1/n\sum _{i\sim j}d_id_j>1/n^2\sum _i d_i \sum _j d_j^2$, we still have similar paradoxes, i.e., the average degree of a neighbor of a neighbor of a random node is larger than the average degree of a random node. If we have the opposite inequality, we see a paradox in a counterintuitive sense in comparison with the friendship paradox. And if we have equality, we do not see any paradox. However, the paradox is always valid for $\ell$ odd since the numerator of Eq. (8) is always negative for $\ell$ odd due to Theorem 4.2 in Ref.^5^ that was originally proposed in Ref.^17^. The theorem says that, given positive integers *r* and *s* such that $r+s$ is even, we have ${\textbf{1}}^TA^r{\textbf{1}}\cdot {\textbf{1}}^TA^{s}{\textbf{1}}\le {\textbf{1}}^TA^{r+s}{\textbf{1}}\cdot {\textbf{1}}^T{\textbf{1}}$.

For a local definition, the equation can be reduced to Eq. (10),

10 $$\begin{aligned} \delta _l^{(\ell )} =\frac{{\textbf{1}}^TA{\textbf{1}}-{\textbf{1}}^TD^{-1}A^{\ell }D{\textbf{1}}}{{\textbf{1}}^T{\textbf{1}}}, \end{aligned}$$

where it can be either positive or negative under different circumstances. Statements similar to the relationship of $\delta _l$ and $\delta _g$ can be derived for this purpose.

Hence, theory suggests that the enmity and friendship paradoxes for higher orders may not always be true and might only hold under some circumstances (e.g., particular sorts of networks, or particular regimes such as related to whether geodesic backsteps are allowed); while we note these mathematical observations here, we leave empirical investigation of these details to future work.

## Methods

Despite the mathematical isomorphism of the enmity paradox and the friendship paradox formulations (in the worlds of positive and negative ties, respectively), the empirical existence and the *strength* of the enmity paradox in real networks is not guaranteed—given the differences between the negative and positive environments. Positivity, for example, is characterized by a large clustering coefficient, reciprocity, and homophily^18,19^, while negativity can even seem like mere noise^18,20^. Here, we characterize some of these structural characteristics of social networks before investigating the enmity paradox, comparing positive and negative ties. These noteworthy differences can be partly explained via the inherent avoidance between the sender and receiver of negative ties, which leads to low information transfer^18^; in other words, people are more likely to know who likes them (because the other persons are more likely to so declare) than they are to know who dislikes them (which is information that is more often kept private)^21^.

Several distinct measures might play an important role in explaining the friendship paradox. (1) We denote the clustering coefficient using $T_l$, which is the local adaptation of the transitivity measure (Supplementary Material, Sect. B), and transitivity as $T_g$. (2) The inversity measure ($H_i$) (which is not the same as degree assortativity, with which it is negatively correlated) has a direct effect on the local friendship paradox strength^12^. (3) The degree assortativity ($H_a$) is also important^14^. (4) A heterogeneity index that captures starlike graphs—similar to the inversity measure—also plays an important role for the severity of friendship paradox. For degree assortativity, we can use extant measures^22^; and for starlike strength, we use a measure that represents topological heterogeneity in complex networks and is maximal for star graphs ($H_{*}$)^23^. We also consider (5) the variance of the degree ($H_{\text{var}}$), and (6) a novel heterogeneity measure ($H_{\mathrm{deg-div}}$) that reflects degree diversity and has a close relationship with entropy^24^. To characterize other differences between positive and negative ties, we can also compare four more topological properties, including the reciprocity, the clustering coefficient, the homophily between the positive and negative ties, and the normalized betweenness-centrality (the mathematical definitions for all these measures are provided in Supplementary Material, Sect. B).

### Maximum paradox strength

The severity of the paradoxes depends on network structure. The global paradox strength is proportional to the variance of its degree sequence divided by its average degree. To maximize the global paradox strength given a constant average degree, we would need to maximize the degree variance by rewiring the edges so that the degree values oscillate between extreme values 1 and $k_{max}$. Similarly, a greedy rewiring algorithm can increase the local paradox strength^12^. A cross-rewiring of paired edges that connect the nodes with extreme degrees—substituting connections of smallest degree nodes with the largest degree nodes instead of connection of medium degree nodes—can increase the strength of the paradox, with star networks having the maximum value. Therefore, the enmity and friendship paradoxes would appear to have the greatest strength in both global and local formulations when connections have a star shape.

## Data

We use data from a sociocentric network study of 24,678 people aged 11 to 93 years (with a mean age of 32) in 176 geographically isolated villages in western Honduras^11^. The Yale IRB and the Honduran Ministry of Health approved all data collection procedures (Protocol # 1506016012), and all participants provided informed consent before enrollment in the study.

**Figure 2:**
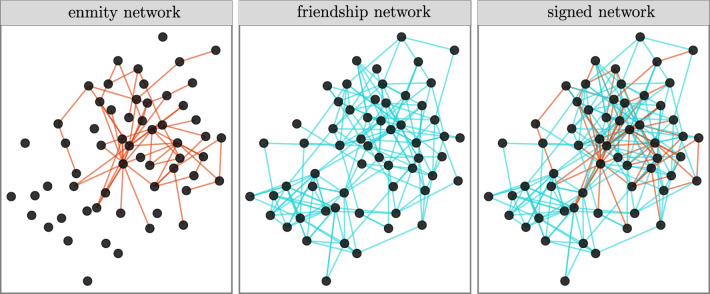
A visualization of the enmity, friendship, and signed networks within one Honduras village. The negative ties tend to be arranged in a star-shaped pattern, whereas the friendship ties contribute more towards larger-scale transitivity. Red indicates negative ties and blue indicates positive ties.

We first construct 176 binary directed signed networks. We use three name generators to determine (potentially overlapping) positive ties (“Who do you spend your free time with?” “Who is your closest friend?” and “Who do you discuss personal matters with?”). And we use one name generator for negative ties (“Who are the people with whom you do not get along well?”). Here, we focus on the enmity paradox for undirected (symmetrized) networks (an example village is illustrated in Fig. 2). Results for undirected (reciprocated) and directed networks are in the Supplementary Material, Sects. C and D, respectively.

## Results

Across the whole population, nodes have an average of 6.89 (${\text{SD}} = 3.79$) friends, and these friends have an average of 8.40 (${\text{SD}} = 2.52$) friends. In order to find the difference at the individual level, we must remove isolated nodes; and the average consequent difference is $- 1.5$ (${\text{SD}} = 3.57$). For the enmity networks, a node has an average of 1.26 (${\text{SD}} = 1.70$) enemies, while these enemies have an average of 3.40 (${\text{SD}} = 2.11$) enemies. After removing the isolated nodes, the average difference between the number of enemies and the number of enemies of enemies is $- 1.10$ (${\text{SD}} = 2.57$). For the remaining analyses, we remove the isolated nodes from the constructed networks.

**Figure 3:**
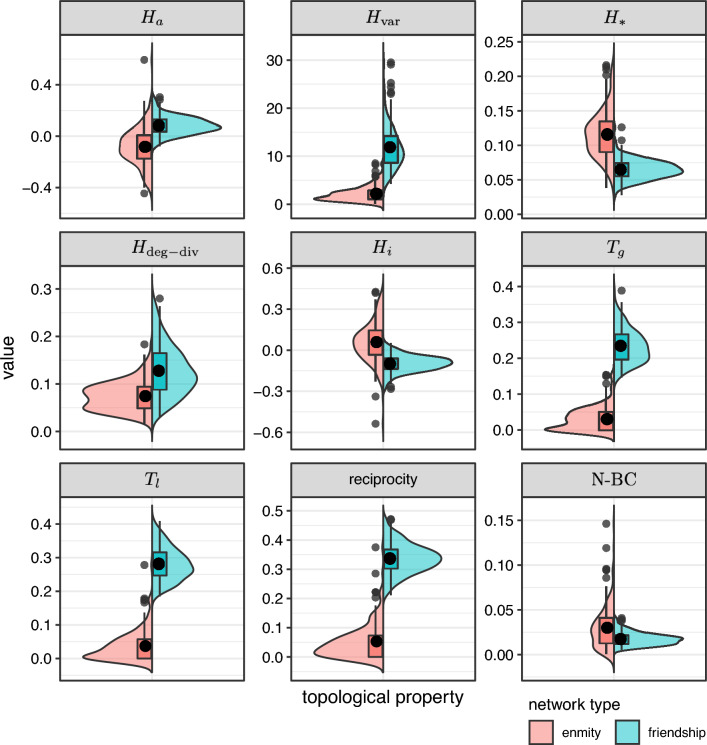
Comparison of topological properties between friendship and enmity networks. $H_a$, $H_{\text{var}}$, $H_{*}$, $H_{\mathrm{deg-div}}$, $H_i$, $T_g$, $T_l$, and ${\mathrm{N-BC}}$ represent degree assortativity, variance, starlike strength, degree-diversity, inversity, transitivity, mean clustering coefficient, and normalized betweenness-centrality, respectively.

Core topological properties of friendship and enmity networks are presented in Fig. 3. The reciprocity of positive edges is much larger than the reciprocity of negative edges, in part due to the relative scarcity of negative ties. Therefore, we expect the undirected enmity networks constructed from reciprocated edges to be much sparser compared to the friendship networks (Supplementary Material, Sect. C). The clustering coefficient of positive edges is much larger than the clustering coefficient of negative edges; that is, friendship networks have more triangles than enmity networks (Fig. 2). The presence of starlike motifs is therefore expected to be more prevalent in negative environments (Fig. 2). This is in turn aligned with the results for both $H_{*}$, and $H_i$. The variance of degree for positive networks is much larger than the negative networks. Thus, it appears that, among the different factors contributing to the strength of the enmity paradox, the starlike indices ($H_i$ and $H_{*}$) align with greater strength, and the lower $H_{\text{var}}$ opposes it. Finally, we also plotted the difference between the normalized betweenness centrality (${\mathrm{N-BC}}$) of the positive and negative worlds to emphasize how the negative world has a more starlike shape as the maximum value of unnormalized ${\text{BC}}$ is achieved by the central point in a star network^25^.

**Figure 4:**
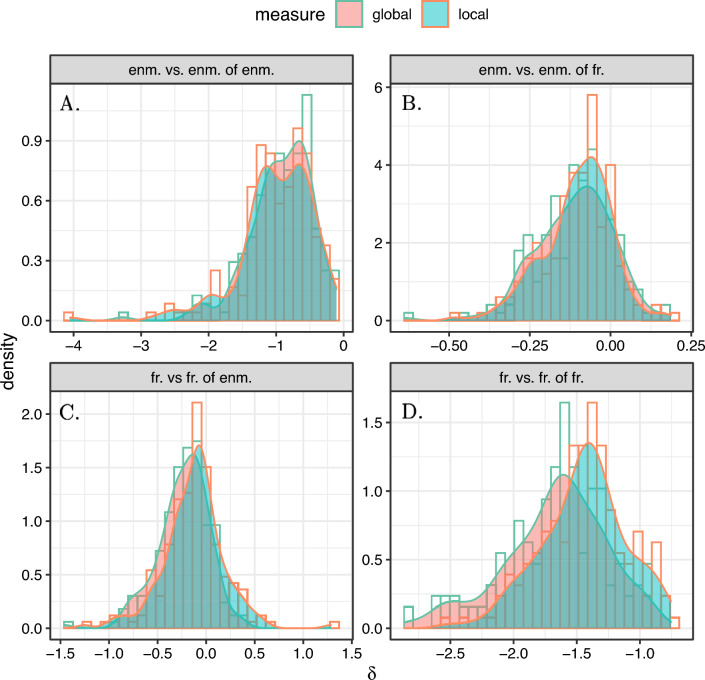
Histograms of $\delta _g$ and $\delta _l$ for undirected (symmetrized) networks among 176 village networks. The histograms of enmity and friendship paradoxes are provided in (**A,D**), respectively. Other panels represent the histograms of mixed-world paradox strengths for the mixed worlds. The histogram in (**B**) shows the global and local paradox distributions for the difference between the number of a person's enemies and the number of enemies of their friends, while (**C**) represents the difference between the number of a person's friends and the number of friends of their enemies.

**Figure 5:**
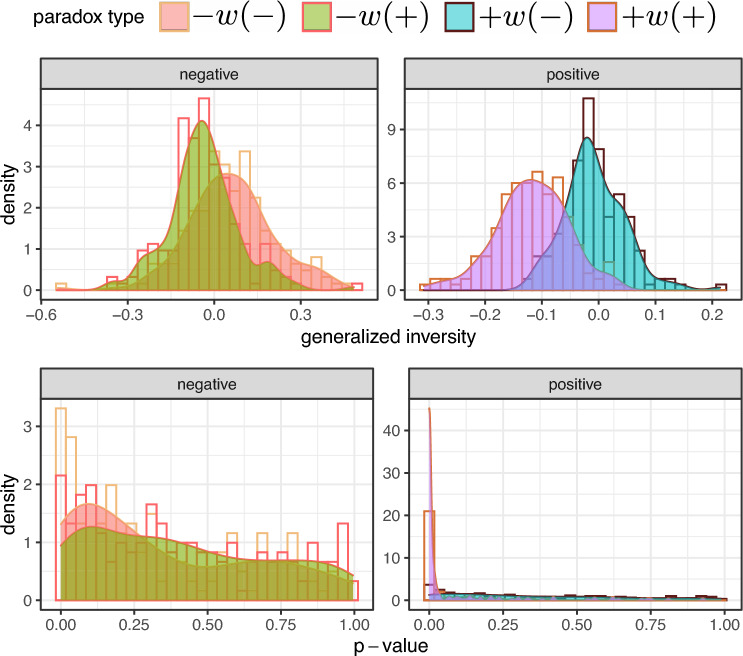
The village-level inversity distribution for undirected (symmetrized) networks. In terms of notation, the $-w$ and $+w$ indicate the type of one’s neighbor, as one’s enemy and friend, respectively. The $(-)$ and $(+)$ denote the type of comparison as enemies or friends. These inversities are aligned with four cases: the enmity paradox in the negative world, $-w(-)$; the friendship paradox in the positive world, $+w(+)$; and the mixed-world paradox in the two mixed worlds, $-w(+)$/$+w(-)$. The distribution of these correlations over 176 village networks in the Honduras dataset is represented in the upper row, while the *P*-values of these correlation tests for these networks are represented in the bottom row. These (correlations, *p*-values) for the whole dataset for the above four cases can be summarized as $(- 0.09,<2.2e^{-16})$, $(- 0.21, <2.2e^{-16})$ for enmity $-w(-)$ and friendship $+w(+)$ paradoxes, and $(-0.09,<2.2e^{-16})$/$(- 0.05, <2.2e^{-16})$ for the mixed-world paradox in the two mixed worlds, when we compare one’s number of friends/enemies with the number of friends/enemies of their enemies/friends $-w(+)$/$+w(-)$. For the friendship paradox $+w(+)$, the distribution of inversity is predominantly distributed over negative values, which results in $\delta _g<\delta _l$, whereas the other distributions are around 0, making the local and global strengths almost identical (Fig. 4).

First, we study the global and local enmity and friendship paradoxes among the 176 villages, and we present the histogram of these values in Fig. 4. We observe large strengths in the enmity paradox and the friendship paradox in negative and positive worlds, respectively, although smaller for the negative world (the 95% confidence intervals are computed using one sample *t*-test and provided in Supplementary Material, Fig. S2). Additionally, given slightly negative $H_i$ values for friendship networks (Fig. 5), we expect the strength of local paradox to be greater than the global paradox for friendship networks as compared to enmity networks (Eq. (5), Fig. 4A,D, Supplementary Material, Fig. 5). The global and local paradoxes for enmity networks and also for the mixed worlds, however, are nearly equivalent, as expected due to their balanced $H_i$ values (Fig. 5). (Details regarding the computation of $H_i$ in the mixed world (the generalized inversity) are provided in Supplementary Material, Sect. G).

The results for the mixed worlds for undirected (symmetrized) networks are also presented in Fig. 4B,C. Although the mixed-world paradox in the mixed world disappears in undirected (reciprocated) networks due to the sparsity of reciprocated negative edges (Supplementary Material, Figs. S1, S2), for undirected (symmetrized) networks, the paradox strengths are significantly larger than 0 (Supplementary Material, Fig. S2). In other words, in undirected networks using symmetrized edges, our friends are more likely to have more enemies than we do (Fig. 4B, Supplementary Material, Fig. S2) and our enemies are more likely to have more friends than we do (Fig. 4C, Supplementary Material, Fig. S2).

**Figure 6:**
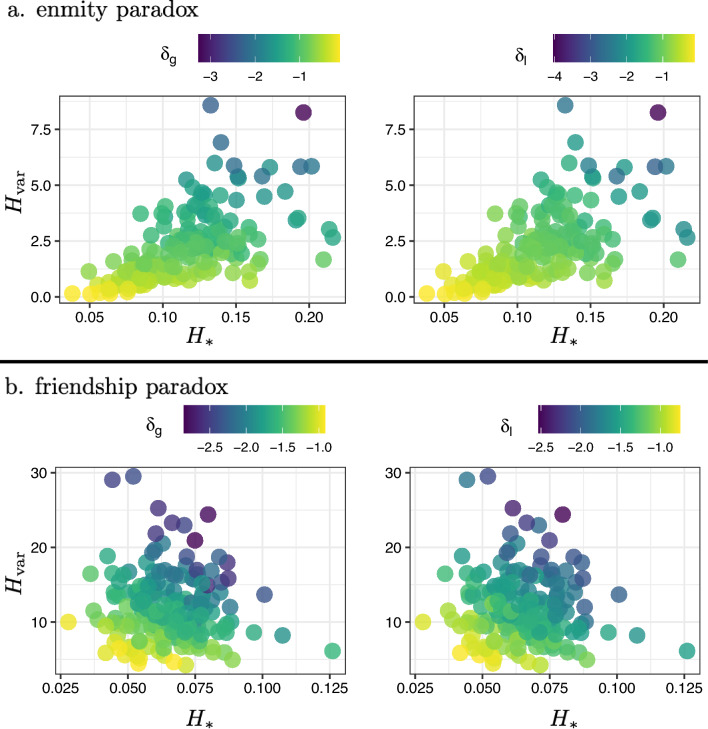
Critical features in enmity and friendship paradoxes. The heat maps show the global and local paradox strengths for undirected (symmetrized) networks as a function of two measures of degree variance $H_{\text{var}}$ and the starlike embedding $H_{*}$. The larger the variance and the more the starlike embedding, the stronger the paradox.

To understand the effect of different topological properties noted in Fig. 3 on the enmity paradox strength, we analyze the relationship between the local and global paradox measures and the topological properties. Using regression models, we can characterize the effect of various topological features on friendship and enmity paradox strengths (Supplementary Material, Sect. E). Among the measures, the degree variance $H_{\text{var}}$ and the starlike embedding $H_{*}$, besides the degree diversity $H_{\mathrm{deg-div}}$ and inversity $H_i$, can explain a significant portion of the intervillage variance in paradox strengths ($R^2\in [0.94,0.97]$; with $\text{N = 176}$ villages). Furthermore, $H_{\text{var}}$ and $H_{*}$ also have large effects. This relationship is illustrated in Fig. 6 (the relationships between the strength and other heterogeneity measures are highlighted in Supplementary Material, Figs. S6, S7). As expected, the larger the variance and the more the starlike embedding, the stronger the paradox.

**Figure 7:**
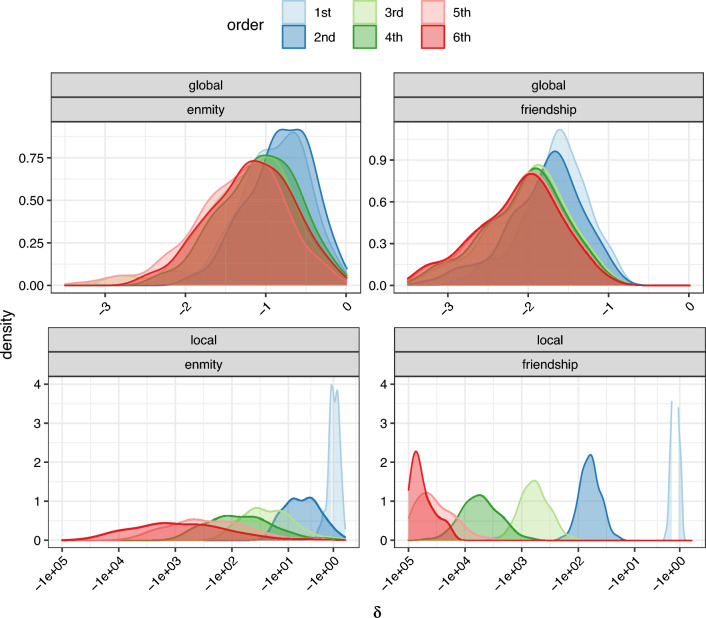
Higher-order enmity and friendship paradox. Paradox strength increases with higher order compared to first order (the figure is plotted for first to sixth order). To better visualize the results of local paradox (bottom row), we employ the signed pseudo-logarithm transformation.

The higher-order enmity and friendship paradoxes are presented in Fig. 7 (for the first to the sixth order). Because high-degree individuals are counted exponentially more than individuals with low degrees when increasing the order of random walks through the network, the higher-order enmity and friendship paradoxes are more severe than the typical enmity and friendship paradoxes. This phenomenon is much more severe in local paradoxes when we compare the degree of a random node with its higher-order neighbors (Fig. 7, bottom row).

**Figure 8:**
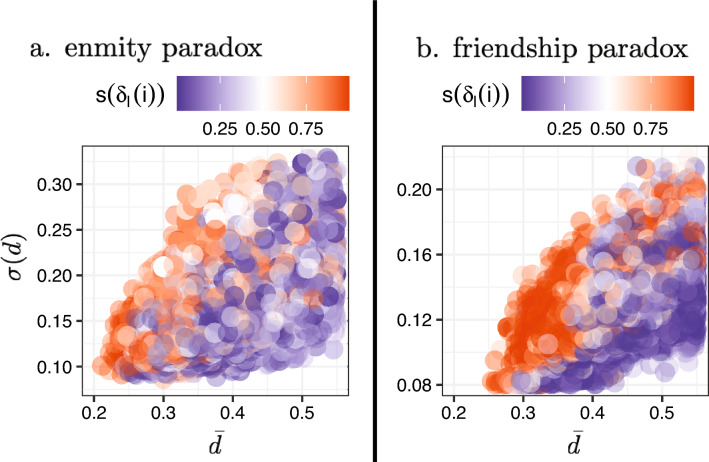
The relationship between a node’s contribution in the local enmity and friendship paradoxes and the location of the nodes within a network. The embedded points in the lower left of the figure correspond to central locations, while points in the upper right are in peripheral locations. Here, we use a sigmoid function $s(x)=1/1+e^{-x}$ in order to increase the small positive and negative differences and reduce the larger differences. The peripheral nodes have a larger paradox strength compared with the central nodes.

To investigate the relationship between a node’s contribution to the local enmity and friendship paradoxes and its topological location, we can plot the nodal contribution versus the location of the nodes in a network. The location of the nodes is defined by using a simple fact in networks, namely that the nodes in the center of a network have a smaller distance from other nodes, while the nodes in the periphery have a larger distance from other nodes. Therefore, if we embed every node using its average and standard deviation of distances from other nodes in a network—normalized by the network diameter within each network component separately—we find that, in a network, peripheral nodes have a greater paradox strength than central nodes (Fig. 8).

Finally, the results for the generalized enmity paradox are provided in Supplementary Material, Sect. F, Tables S4, S5, Figs. S9 and S10. The results document the existence, for instance, of the generalized paradoxes in both enmity and friendship with respect to wealth. Due to the higher correlation between non-topological features and positive degree, the generalized friendship paradox is stronger than the generalized enmity paradox.

The results for undirected (reciprocated) and directed networks are provided in Supplementary Material, Figs. S1 and S4, respectively. For undirected (reciprocated) networks, the enmity and mixed-world paradox strengths in the negative world and the two mixed worlds, respectively, are significantly smaller compared to undirected (symmetrized) networks (Supplementary Material, Fig. S2)—due to the small reciprocity in enmity networks. Regarding directed networks, we observe that the global enmity paradox strength is usually greater than the corresponding local strength in a purely negative world (the unpaired *t*-tests for comparison of global and local paradox strengths are provided in Table S2). Interestingly, the enmity paradox for directed negative networks conveys contradictory information. Negative values of the global paradox strength $\delta _g$ represent the enmity paradoxes on a global scale, whereas positive values of the local paradox strength $\delta _l$ indicate that the paradoxes are in the opposite direction locally (more details in the Supplementary Material, Sect. D). The difference between local and global enmity paradox strengths for directed networks can be explained using an inversity measure (Supplementary Material, Sects. D, G, Fig. S13).

## Discussion

Our perception of the world can be distorted by the sampling bias towards high-degree nodes that arise as a result of the friendship paradox^1^. In our minds, if not in reality, our friends have more friends, our collaborators have more collaborators, and our colleagues are wealthier or happier than we ourselves are. Here, we have shown empirically that the same observations hold with respect to antagonistic ties. Yet, with a lower clustering coefficient, lower degree assortativity, and greater inversity, we expect even more starlike shapes in enmity networks. These factors all make the enmity paradox even more probable than the friendship paradox. Moreover, the enmity paradox obtains in a negative world when comparing the number of an ego’s enemies with their enemies’ enemies as well as in mixed worlds when we compare an ego’s friends with the ego’s enemies’ friends and an ego’s enemies with an ego’s friends’ enemies.

Our approach to, and equations regarding, the mixed world have the versatility to be effectively implemented in multiplex networks that consist of multiple layers of types of ties more generally. That is, the argument generalizes to the case of any network with two (or more) distinct edge types (not just friends/enemies, but also friends/family, emotional/physical sources of support, and so on). Consequently, we can investigate paradoxes that emerge between the layers of such networks. It is noteworthy that a positive correlation between the degrees across the different layers is essential for such paradoxes (alter-biased perception) to surface. If the correlation between layers is small, there is no difference between the perceptions coming from different layers. Of course, a negative correlation between layers can support paradoxes in the opposite direction.

Some topological differences between enmity and friendship networks (e.g., the smaller variance and smaller reciprocity in enmity networks) suggest possibly divergent perceptional biases. For example, since the paradox strength is proportional to the degree variance, it could be lower for enmity networks; however, we observe similar paradox patterns in both enmity networks and in a mixed world of friendship and antagonistic ties.

There is also a fundamental connection between the generalized friendship paradox (involving non-topological features) and the enmity paradox. If we treat the number of enemies a person has as simply a non-topological attribute for the *positive* network, then the enmity paradox just follows from the generalized friendship paradox. Looking at the correlation between positive and negative degrees reveals these observations (Supplementary Material, Fig. S11). As a result of the positive correlation between positive and negative degrees in most of the village networks ($\text {Pearson's correlation} = 0.18$, $p<10^{-16}$)—in other words, the empirical reality that people who have many friends also tend to have more enemies—we are able to actually answer the question of why the enmity paradox exists in the first place through the lens of the generalized friendship paradox.

These paradoxes have further implications. Our understanding of social norms and of our social standing is influenced by our perceptions of those around us. For instance, the friendship paradox can help explain systematic biases in social perceptions such as regarding the prevalence of binge drinking and risky behaviors^16,26^. Furthermore, the friendship paradoxes can explain why a given behavior in a society can be amplified. This can occur in two interwoven phases, where popular individuals act more intensely for activities associated with strategic complementarities, and those who are prone to certain behaviors interact more with other people who are also involved in that behavior, amplifying the effects of this behavior^16^. As a result, perceptions of behavior increase, and this could contribute to an increase in the behavior along the lines of the perception. Misperceptions about the habits of one’s enemies could act similarly.

Such inter-personal influence, even if biased, can in turn be exploited to foster cascades, as field experiments have shown^10,11,13,27^. Indeed, as a result of phenomena like the friendship and enmity paradoxes, we could further perfect network targeting algorithms that exploit the friendship paradox; and taking into account a person’s enemies could make network targeting even more effective.

Biases associated with our antagonistic ties could be consequential in still another way. For instance, the friendship paradox may intensify homophilous patterns in network formation due to the misperception^16,28^. Because of these paradoxes and the misperceptions they can give rise to, people might form a miscalbirated assessment of their own attributes and thus seek out connections with people different than they might otherwise truly wish or “deserve.” The enmity paradox might similarly change our perceptions of reality and may function as a deterrent force in the formation of network ties between a person and the social connections around a person’s antagonists. When people judge whom to either befriend or avoid, they may be biased in ways that might harm their interests.

### Supplementary Information

Supplementary Information.

## Data Availability

The full datasets generated and/or analyzed during the current study are not publicly available due to several considerations, including the commitments to the research subjects and the sensitive nature of the health and social data in these small communities that could potentially allow decryption or individual identification, but they are available from the corresponding author on reasonable request, and subject to a DUA. The replication code and the network data for a sample of 22 signed villages are provided at https://github.com/Aghasemian/EnmityParadox.
