# Supplementary material for: The enmity paradox
Source: Sci Rep. 2023 Nov 16;13:20040. doi: 10.1038/s41598-023-47167-9 (PMC10654772; doi:10.1038/s41598-023-47167-9)
Supplement: Supplementary file 1 — Supplementary Information. [file 41598_2023_47167_MOESM1_ESM.pdf]

Supplementary Material
The Enmity Paradox

Amir Ghasemian[1] and Nicholas A. Christakis[1]

[1] *Yale Institute for Network Science, Yale University, New Haven, CT 06511, USA*

Section A: Data

Our data come from wave 1 of a sociocentric network study of 24,678 people aged 11 to 93 years (with a mean age of 32) in 176 geographically isolated villages in western Honduras [1]. Using this empirical data, we first construct 176 binary directed signed networks (with no multi-edges or self-loops). We use three name generators to determine (overlapping) positive ties ("Who do you spend your free time with?" "Who is your closest friend?" and "Who do you discuss personal matters with?") to construct the positive world; and we used one name generator for negative ties ("Who are the people with whom you do not get along well?") to construct the negative world. Due to the village-based nature of our analysis, we also exclude the uncommon connections outside of the villages.

The reciprocity of negative ties in the analyzed dataset is much smaller than that of positive ties, which may influence the findings. There are several factors contributing to this, including self-censorship by individuals who do not share sensitive information in order to avoid embarrassment, to reduce the risk of social norm violation, and to protect their privacy [2, 3]. In addition, there can be a lack of information flow due to the avoidance of any interaction with the receiver of the antagonistic tie by the sender, which may result in the receiver being unaware of the tie and thus unable to "reciprocate" it [4]. Nevertheless, the amount of reciprocity depends on the question, and studying undirected networks in the Honduras data can be justified based on the fact that negative ties are created from the question "Who are the people with whom you do not get along well?" that is a symmetric relationship, at least in practice. We turned the directed networks into undirected ones by either (1) removing unreciprocated edges or (2) symmetrizing them. Further approaches are described below.

Section B: Measures

1. Inversity measure (H_i): this measure is proposed in [5] where it has been shown that it determines the relative effectiveness of the local and global strategies. Actually, the authors have shown that $\mu_L = \mu_G + H_i\Psi(\kappa_{-1}, \kappa_1, \kappa_2, \kappa_3)$, where, μ_G and μ_L are the global and local mean number of neighbor's enemies/friends, respectively, $\kappa_m = {}^1\!/_n \sum_i k_i^m$ is the m-th moment degree, and $H_i = Cor(k_i, {}^1\!/_{k_j})$, $\forall(i,j) \in E$, i.e., the edge-based correlation of the degree of an endpoint of an edge E with the inverse degree of another endpoint j of that edge is the inversity measure.

2. Degree assortativity (H_a): this measure of homophily measures the tendency for vertices with similar degrees to connect with each other. In [6], the authors have shown that this measure has a key role in the strength of the local definition of the friendship paradox. For degree assortativity, we use the definition in [7], i.e., $H_a = \sum_{j,k} {}^{jk(e_{jk}-q(j)q(k))}\!/_{\sigma_q^2}$, where $q(k)$ is the probability of a random endpoint of a random edge has degree k, and e_{jk} is the probability of a random edge has endpoints of degree j and k. This definition is provided for undirected networks and it can be easily generalized for directed networks.

3. Starlike strength (H_*): this measure is introduced in Ref. [8] and it represents topological heterogeneity in complex networks; it is maximal for star graphs. The normalized heterogeneity index can be written as $H_* = \sum_{(i,j)\in E} \left(k_i^{-1/2} - k_j^{-1/2}\right) / \left(n - 2\sqrt{n-1}\right)$, where n is the number of nodes in a network.

4. Variance of the degree (H_{var}): this typical degree heterogeneity measure is the variance of the degree distribution in a network.

5. Degree-diversity ($H_{\text{deg-div}}$): this is a novel degree heterogeneity measure proposed in [9] that reflects the degree diversity and has a close relationship with the entropy of the degree distribution. Given a network G, the authors define a heterogeneity index h for a network of n nodes as $h^2 = {}^1\!/_n \sum_{k_{\min}}^{k_{\max}} (1 - P(k))^2$, where $P(k)$ is the probability of a random node with degree k, $k_{\min}$ and $k_{\max}$ are the minimum and maximum degrees, respectively, and the summation is only for k, which $P(k) \neq 0$. Then, for a completely homogeneous network of

k-regular network, it is the case that $h_{\text{hom}} = 0$, and for the most heterogenous case, where the $P(k)$ is uniform, $h_{\text{het}} = 1 - {}^{3}/_{n} + {}^{(n+2)}/_{n^3}$. Therefore, the degree diversity is the normalized version of h as $H_{\text{deg-div}} = {}^{h}/_{h_{\text{het}}}$.

6. Transitivity T_g and clustering coefficient T_l: transitivity is one of the two most common topological features that is designed to measure the commonness of triangles in social networks. This global measure computes the fraction of transitive triads that are subgraphs of triangles [10]. Another topological feature, the clustering coefficient, which is very similar to transitivity, computes the fraction of triads that are subgraphs of triangles for each node i, and then averages these values. As a result, we can consider the clustering coefficient as the local adaptation of the transitivity measure, and we refer to the clustering coefficient as T_l, and transitivity as T_g. In most graphs, these two measures take similar values; however, they are rare occasions that they disagree.

7. Normalized betweenness-centrality ($\mathrm{N-BC}$): the number of shortest paths traversing a node v represents its importance in information flow in a network and is called betweenness centrality (BC). The maximum value of this measure is achieved by the central point in a star, and it is ${}^{n^2-3n+2}/_{2}$ [11]. Therefore, the $\mathrm{N-BC}$ is a normalization of BC by this maximum value.

Section C: Enmity paradox in undirected networks

The generalized inversity in mixed worlds

Based on the inversity measure for undirected networks, it is possible to formalize the differences between global and local paradoxes in four different scenarios introduced in the main text: (1) the enmity paradox; (2) the friendship paradox; and the mixed-world paradox in the two mixed worlds of: (3) when comparing the number of ego's friends with the number of ego's enemies' friends; and 4. when comparing the number of ego's enemies with the number of ego's friends' enemies.

Here, we summarize these equations in the following manner. (Detailed proof of the generalization of the inversity measure for the mixed worlds (Eqs. S3 and S4) are provided in Section G.)

1.
$$\delta_{g,-w}(-) - \delta_{l,-w}(-) \propto cor(k_{(-),i}, {}^{1}/_{k_{(-),j}} | (i,j) \in E_{(-)}) \tag{S1}$$

2.
$$\delta_{g,+w}(+) - \delta_{l,+w}(+) \propto cor(k_{(+),i}, {}^{1}/_{k_{(+),j}} | (i,j) \in E_{(+)}) \tag{S2}$$

3.
$$\delta_{g,-w}(+) - \delta_{l,-w}(+) \propto cor(k_{(+),i}, {}^{1}/_{k_{(-),j}} | (i,j) \in E_{(-)}) \tag{S3}$$

4.
$$\delta_{g,+w}(-) - \delta_{l,+w}(-) \propto cor(k_{(-),i}, {}^{1}/_{k_{(+),j}} | (i,j) \in E_{(+)}) \tag{S4}$$

Enmity paradox in undirected (reciprocated) networks

The results for undirected (symmetrized) networks have been provided in the main text. Here, the results for undirected networks constructed by only keeping the reciprocated edges are presented (see Fig. S1). Once the isolated nodes are removed, many undirected (reciprocated) networks disappear or become very small, and the results are limited to networks with at least 8 nodes (27 out of 176 networks). Additionally, due to the small reciprocity in the negative world, the enmity paradox for undirected enmity networks constructed with reciprocated edges is much smaller than the friendship paradox for friendship networks constructed similarly (see Fig. S1, A versus D). Essentially, this observation reflects the sparsity of the networks created as a result of antagonistic ties being infrequently reciprocal compared to positive ones. The amount of reciprocity varies from question to question, and we believe that questions such as "Who are the people with whom you do not get along well" should be accompanied by greater reciprocity. Therefore, it is possible that measurement errors and self-censorship lead to smaller reciprocity, further justifying symmetrized undirected networks in our study.

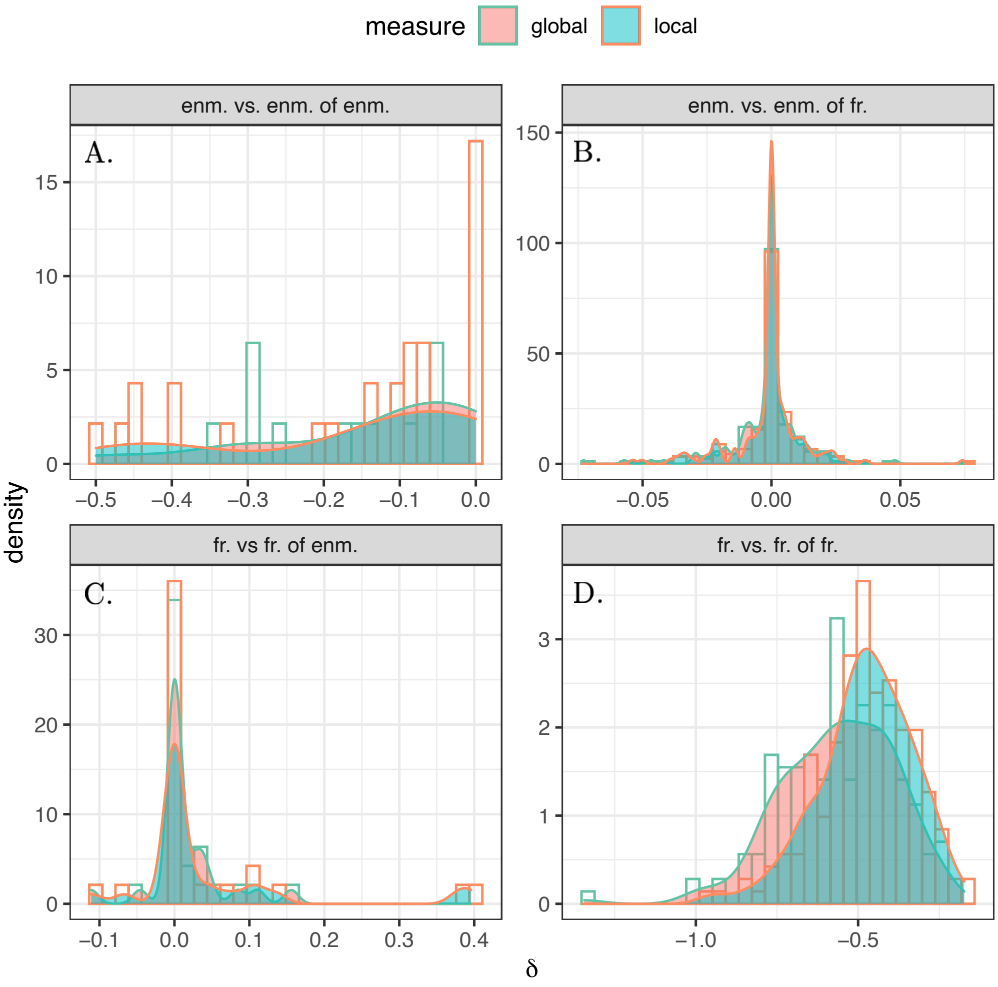

FIG. S1. Histograms of δ_g and δ_l for undirected (reciprocated) networks. The histograms of enmity and friendship paradoxes are provided in panels A and D, respectively. Other panels represent the histograms of mixed-world paradox strengths for the mixed worlds. The histogram in panel B shows the global and local paradox distributions for the difference between the number of our enemies and the number of enemies of our friends, while panel C represents the difference between the number of our friends and the number of friends of our enemies. These paradoxes are minimized in undirected (reciprocated) networks when compared to undirected (symmetrized) networks (see the main text), especially in mixed worlds due to the sparsity of negative edges that cause the networks created to be very small after removing isolated nodes (only 27 out of 176 networks were left in our analysis).

The results for the mixed worlds in undirected (reciprocated) networks are also presented in Fig. S1. As with the enmity paradox of undirected networks constructed with reciprocated edges, we do not observe the mixed-world paradox in the mixed worlds with reciprocated edges due to the sparsity of reciprocated negative edges; however, for undirected (symmetrized) networks (main text, Fig. 4) the paradoxes are significantly greater than for undirected (reciprocated) networks (Fig. S2).

Section D: Enmity paradox in directed networks

Here, we derive the equations of the enmity paradox for directed networks. We follow a similar convention as in Ref. [12] and use distinguishing names for alters who introduce egos as antagonistic/friendship connections and alters that egos introduce as antagonistic/friendship connections. We may name those whom a person reports as antagonistic/friendship connection as “enemy”/“friend” and those who introduce a person as antagonistic/friendship connection as “hater”/“liker.”

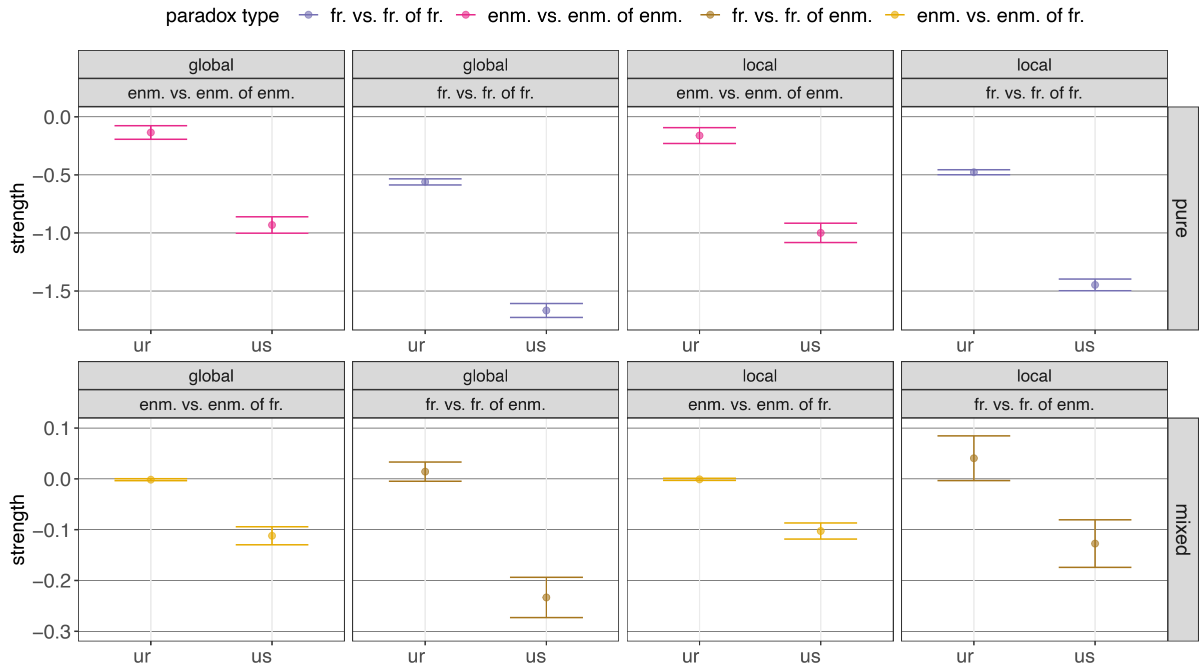

FIG. S2. The 95% confidence intervals of δ_g and δ_l for undirected (reciprocated) (ur) and undirected (symmetrized) (us) networks (calculated using one sample t-test). The enmity, mixed-world, and friendship paradox strengths are significantly smaller for ur networks due to the smaller reciprocity of negative ties.

Then, we have four possibilities that we should consider (Fig. S3): 1. our enemies/friends have more haters/likers than we do; 2. our haters/likers have more enemies/friends than we do; 3. our enemies/friends have more enemies/friends than we do; and 4. our haters/likers have more haters/likers than we do. Similar to what the authors in Ref. [12] have shown for the positive world of followers/followees, the first two statements are correct mathematically, but, for the last two, a positive correlation between the in-degree and out-degree is required.

The global formulation of the enmity paradox for directed networks is a comparison between the average in-degree/out-degree of a random node i and the average in-degree/out-degree of its neighbors' in-degrees/out-degrees when the neighbor is chosen between the enemies and haters as described below. Here, $A_{i,j} = 1$ means node j is an enemy of node i and node i is a hater of node j. In other words, node i reports node j as its antagonistic connection. As a result of the symmetry, the equations for the directed friendship paradoxes are similar. To simplify the notation, we use A instead of $A_{(-)}$. As we did for the undirected networks in the main text, these equations can be generalized to the mixed world when positive and negative ties coexist.

1. the average number of enemies of a random node versus the average number of enemies of a random hater.

$$\delta_{g,in-w}(out) = \frac{(\mathbf{1}^T A \mathbf{1})^2 - \mathbf{1}^T A^T A \mathbf{1} \cdot \mathbf{1}^T \mathbf{1}}{\mathbf{1}^T A \mathbf{1} \cdot \mathbf{1}^T \mathbf{1}} \tag{S5}$$

In this notation, the $in - w$ and $out - w$ indicate the direction of one's neighbor, as one's in-neighbor and out-neighbor, respectively. The (in) and (out) denote the type of comparison as in-degree or out-degree.

2. the average number of haters of a random node versus the average number of haters of a random enemy.

$$\delta_{g,out-w}(in) = \frac{(\mathbf{1}^T A \mathbf{1})^2 - \mathbf{1}^T A A^T \mathbf{1} \cdot \mathbf{1}^T \mathbf{1}}{\mathbf{1}^T A \mathbf{1} \cdot \mathbf{1}^T \mathbf{1}} \tag{S6}$$

3. the average number of enemies of a random node versus the average number of enemies of a random enemy.

$$\delta_{g,out-w}(out) = \frac{(\mathbf{1}^T A \mathbf{1})^2 - \mathbf{1}^T A^2 \mathbf{1} \cdot \mathbf{1}^T \mathbf{1}}{\mathbf{1}^T A \mathbf{1} \cdot \mathbf{1}^T \mathbf{1}} \tag{S7}$$

4. the average number of haters of a random node versus the average number of haters of a random hater.

$$\delta_{g,in-w}(in) = \frac{(\mathbf{1}^T A\mathbf{1})^2 - \mathbf{1}^T A^2\mathbf{1} \cdot \mathbf{1}^T\mathbf{1}}{\mathbf{1}^T A\mathbf{1} \cdot \mathbf{1}^T\mathbf{1}} \tag{S8}$$

The global formulation of the last two scenarios is the same due to symmetry. It is not different when we compute the average number of haters of our haters or the average number of enemies of our enemies globally.

However, it matters when we have the local formulation. For the local formulation, we have again four possibilities (Fig. S3). The in-degree and out-degree vectors are denoted as d_{in} and d_{out}, while the diagonal in-degree and out-degree matrices are denoted as D_{in} and D_{out}, respectively. Therefore, $d_{\text{out},i}$ is the i-th entry of the out-degree vector d_{out}, and, similarly, it can be defined for the in-degree vector d_{in}.

1. the average difference of the number of enemies of a random node with the average number of enemies of its haters.

$$\delta_{l,in-w}(out) = \frac{\mathbf{1}^T A\mathbf{1} - \mathbf{1}^T D_{\text{in}}^{-1} A^T D_{\text{out}}\mathbf{1}}{\mathbf{1}^T\mathbf{1}} \tag{S9}$$

2. the average difference of the number of haters of a random node with the average number of haters of its enemies.

$$\delta_{l,out-w}(in) = \frac{\mathbf{1}^T A^T\mathbf{1} - \mathbf{1}^T D_{\text{out}}^{-1} A D_{\text{in}}\mathbf{1}}{\mathbf{1}^T\mathbf{1}} \tag{S10}$$

3. the average difference of the number of enemies of a random node with the average number of enemies of its enemies.

$$\delta_{l,out-w}(out) = \frac{\mathbf{1}^T A\mathbf{1} - \mathbf{1}^T D_{\text{out}}^{-1} A D_{\text{out}}\mathbf{1}}{\mathbf{1}^T\mathbf{1}} \tag{S11}$$

4. the average difference of the number of haters of a random node with the average number of haters of its haters.

$$\delta_{l,in-w}(in) = \frac{\mathbf{1}^T A^T\mathbf{1} - \mathbf{1}^T D_{\text{in}}^{-1} A^T D_{\text{in}}\mathbf{1}}{\mathbf{1}^T\mathbf{1}} \tag{S12}$$

In the local formulation, $\mathbf{1}^T A\mathbf{1} = \mathbf{1}^T A^T\mathbf{1} = \sum_i d_{\text{out},i} = \sum_i d_{\text{in},i}$. However, to compute the individual values of the vector of average difference we need to write it this way.

1. the difference between the number of enemies of a random node with the average number of enemies of its haters.

$$\Delta_{l,in-w}(out) = (A - D_{\text{in}}^{-1} A^T D_{\text{out}})\mathbf{1} \tag{S13}$$

2. the difference between the number of haters of a random node with the average number of haters of its enemies.

$$\Delta_{l,out-w}(in) = (A^T - D_{\text{out}}^{-1} A D_{\text{in}})\mathbf{1} \tag{S14}$$

3. the difference between the number of enemies of a random node with the average number of enemies of its enemies.

$$\Delta_{l,out-w}(out) = (A - D_{\text{out}}^{-1} A D_{\text{out}})\mathbf{1} \tag{S15}$$

4. the difference between the number of haters of a random node with the average number of haters of its haters.

$$\Delta_{l,in-w}(in) = (A^T - D_{\text{in}}^{-1} A^T D_{\text{in}})\mathbf{1} \tag{S16}$$

The generalized inversity in directed networks

Based on the inversity measure expanded for directed networks, it is possible to formalize the differences between the four pairs of global and local paradoxes in directed networks [12]. Here, we summarize these equations in the following manner. (Detailed proof of the generalization of the inversity measure for directed networks is provided in Section G.)

1.

$$\delta_{g,in-w}(out) - \delta_{l,in-w}(out) \propto cor(k_{i,out}, {}^{1}/_{k_{j,in}} | (i,j) \in E) \tag{S17}$$

2.

$$\delta_{g,out-w}(in) - \delta_{l,out-w}(in) \propto cor({}^{1}/_{k_{i,out}}, k_{j,in} | (i,j) \in E) \tag{S18}$$

3.

$$\delta_{g,out-w}(out) - \delta_{l,out-w}(out) \propto cor({}^{1}/_{k_{i,out}}, k_{j,out} | (i,j) \in E) \tag{S19}$$

4.

$$\delta_{g,in-w}(in) - \delta_{l,in-w}(in) \propto cor(k_{i,in}, {}^{1}/_{k_{j,in}} | (i,j) \in E) \tag{S20}$$

Similarly, we can easily generalize the equations corresponding to the local and global formulations of enmity and friendship paradoxes in directed networks to the mixed world of directed networks, when both antagonistic and friendship interactions coexist. For example, we can compare the number of enemies of a random node with the number of enemies of a random liker in the global and local formulations as $\delta_g = \left(\mathbf{1}^T A_{(-)}\mathbf{1}\cdot\mathbf{1}^T A_{(+)}\mathbf{1} - \mathbf{1}^T A_{(+)}^T A_{(-)}\mathbf{1}\cdot\mathbf{1}^T\mathbf{1}\right)/\mathbf{1}^T A_{(+)}\mathbf{1}\cdot\mathbf{1}^T\mathbf{1}$ and $\delta_l = \left(\mathbf{1}^T A_{(-)}\mathbf{1} - \mathbf{1}^T D_{(+),\text{in}}^{-1} A_{(+)}^T D_{(-),\text{out}}\mathbf{1}\right)/\mathbf{1}^T\mathbf{1}$, respectively. However, a careful study of these mixed worlds for directed networks is out of scope of this study, though it could be examined in future work.

Also for the generalized enmity paradox, we can expand the equations for directed networks. The global formulation of the generalized paradox for directed networks can be written for enemies in Eq. S21, and for haters in Eq. S22.

$$\delta_g(x) = \frac{\mathbf{1}^T D_x \mathbf{1}\mathbf{1}^T A_{(-)}\mathbf{1} - \mathbf{1}^T A_{(-)} D_x \mathbf{1}\cdot\mathbf{1}^T\mathbf{1}}{\mathbf{1}^T A_{(-)}\mathbf{1}\cdot\mathbf{1}^T\mathbf{1}} \tag{S21}$$

$$\delta_g(x) = \frac{\mathbf{1}^T D_x \mathbf{1}\mathbf{1}^T A_{(-)}\mathbf{1} - \mathbf{1}^T A_{(-)}^T D_x \mathbf{1}\cdot\mathbf{1}^T\mathbf{1}}{\mathbf{1}^T A_{(-)}\mathbf{1}\cdot\mathbf{1}^T\mathbf{1}}\,. \tag{S22}$$

Similarly, for the local formulation of the generalized paradox, we can write the equations for enemies and haters in Eqs. S23 and S24, respectively.

$$\delta_l(x) = \frac{\mathbf{1}^T D_x \mathbf{1} - \mathbf{1}^T D_{(-),\text{out}}^{-1} A_{(-)} D_x \mathbf{1}}{\mathbf{1}^T\mathbf{1}} \tag{S23}$$

$$\delta_l(x) = \frac{\mathbf{1}^T D_x \mathbf{1} - \mathbf{1}^T D_{(-),\text{in}}^{-1} A_{(-)}^T D_x \mathbf{1}}{\mathbf{1}^T\mathbf{1}}\,. \tag{S24}$$

As part of an analysis of both directed and undirected village networks in western Honduras, we compared the average degrees of all nodes with the average degrees of their neighbors in Table S1. We define neighbors in directed networks in two ways: in-neighbors and out-neighbors. A comparison of the average degrees of the nodes is made with their neighbors' average degrees. We consider four cases for directed networks corresponding to the scenarios presented in Fig. S3.

The empirical results for the aforementioned enmity paradox in directed networks, introduced by four different formulations in Eqs. S5-S12, and their corresponding friendship paradoxes have been provided in Fig. S4. The results indicate that we see global paradoxes for both enmity and friendship directed networks. Also, the strength of these paradoxes is maximum when comparing our number of haters with the number of haters of our enemies and our number of likers with the number of likers of our friends (Table S2), as these two are mathematically supported. Thus, our

value	enmity		friendship	
	node	neighbor	node	neighbor
**(undirected)**				
degree	1.26	3.40	6.89	8.40
**(directed)**				
1. out-degree (in-neighbor)	0.65	2.45	4.13	5.19
2. in-degree (out-neighbor)	0.65	3.04	4.13	6.43
3. out-degree (out-neighbor)	0.65	0.89	4.13	4.30
4. in-degree (in-neighbor)	0.65	0.84	4.13	4.14

TABLE S1. Comparing the average degrees of nodes in 176 village networks in western Honduras with the average degrees of their neighbors for both directed and undirected networks. Directed networks define neighbors as either in- or out-neighbors. We compare the average degree of the nodes with the average degree of their neighbors. For directed networks, four scenarios are considered, corresponding to those presented in Fig. S3.

enemies have more haters than we do, and our friends have more likers. As with the previous two mathematical facts, our likers have more friends than we do, and our haters have more enemies than we do, but the strength of these two mathematical paradoxes is smaller than those from the previous two facts (Table S2). In order for the other four global paradoxes to be valid, the in- and out-degrees must be positively correlated [12]. In view of the positive correlation between in- and out-degrees (Fig. S12), we expect the other four global paradoxes to be satisfied as shown in Fig. S4. Alternatively, while there are local paradoxes when comparing our number of haters/likers with the number of haters/likers of our enemies/friends and also comparing our number of enemies/friends with the number of enemies/friends of our haters/likers, they do not exist or exist in a counterintuitive sense when we compare our number of enemies/friends with the number of enemies/friends of our enemies/friends, or when we compare our number of haters/likers with the number of haters/likers of our haters/likers. In Table S2, we present a comparison of global and local paradox strengths in directed networks in Honduras using unpaired t-tests. Generally, the difference between global and local paradoxes can be explained using the generalized inversity measures for directed networks. The correlations and P-values for the whole dataset are summarized for both enmity and friendship networks in the caption of Fig. S4. Also, a detailed analysis of these correlations has been provided in Fig. S13. Several applications can benefit from exploring the enmity paradox in directed mixed networks including both positive and negative ties. Future research can examine the details of the enmity paradox in such an environment, as well as the relationship between the paradox strength and the topological features of directed networks.

panel in	paradox type	mean		mean difference	P-value	significant
Fig. S4		global δ_g	local δ_l	$\Delta_{gl} = \delta_g - \delta_l$		
A	enm. vs. enm. of enm.	-0.11	1.02	-1.12	0.00	***
B	enm. vs. enm. of htr.	-1.62	-1.60	-0.02	0.51	
C	fr. vs. fr. of fr.	-0.26	-0.06	-0.20	0.00	***
D	fr. vs. fr. of lkr.	-1.11	-0.97	-0.14	0.00	***
E	htr. vs. htr. of enm.	-2.00	-1.98	-0.02	0.85	
F	htr. vs. htr. of htr.	-0.11	0.99	-1.09	0.00	***
G	lkr. vs. lkr. of fr.	-2.37	-2.21	-0.16	0.02	*
H	lkr. vs. lkr. of lkr.	-0.26	0.29	-0.55	0.00	***

$^{***}p < 0.001$; $^{**}p < 0.01$; $^{*}p < 0.05$

TABLE S2. The comparison of global and local paradox strengths in 176 directed network villages in Honduras using unpaired t-tests. The mean difference indicates the average difference between the global and local groups, i.e., $\Delta_{gl} = \delta_g - \delta_l$. The histograms of δ_g and δ_l for all paradoxes in directed networks among 176 network villages are presented in Fig. S4. The "significant" column indicates that the difference between global and local strengths is significant, except for the comparison between the number of one's enemies and the number of enemies of one's haters and between the number of one's haters and the number of haters of one's enemies.

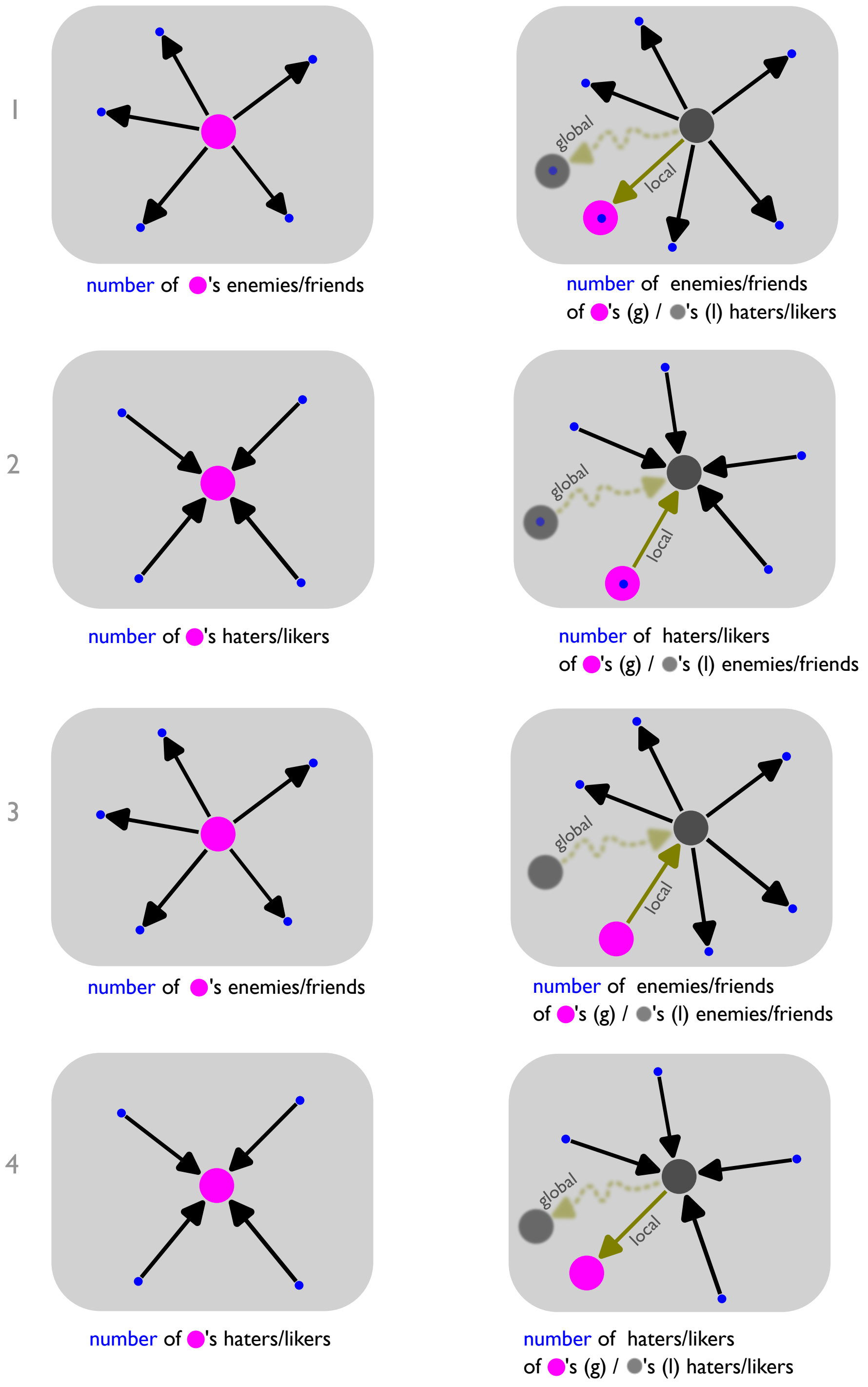

FIG. S3. A schematic of four possibilities in the global and local formulations of the enmity and friendship paradoxes. For local paradoxes, we compare a person's number of in-/out-neighbors with their in-/out-neighbor's average number of in-/out-neighbors. For the global paradoxes, we compare a person's number of in-/out-neighbors with the average number of in-/out-neighbors of a random endpoint of an edge.

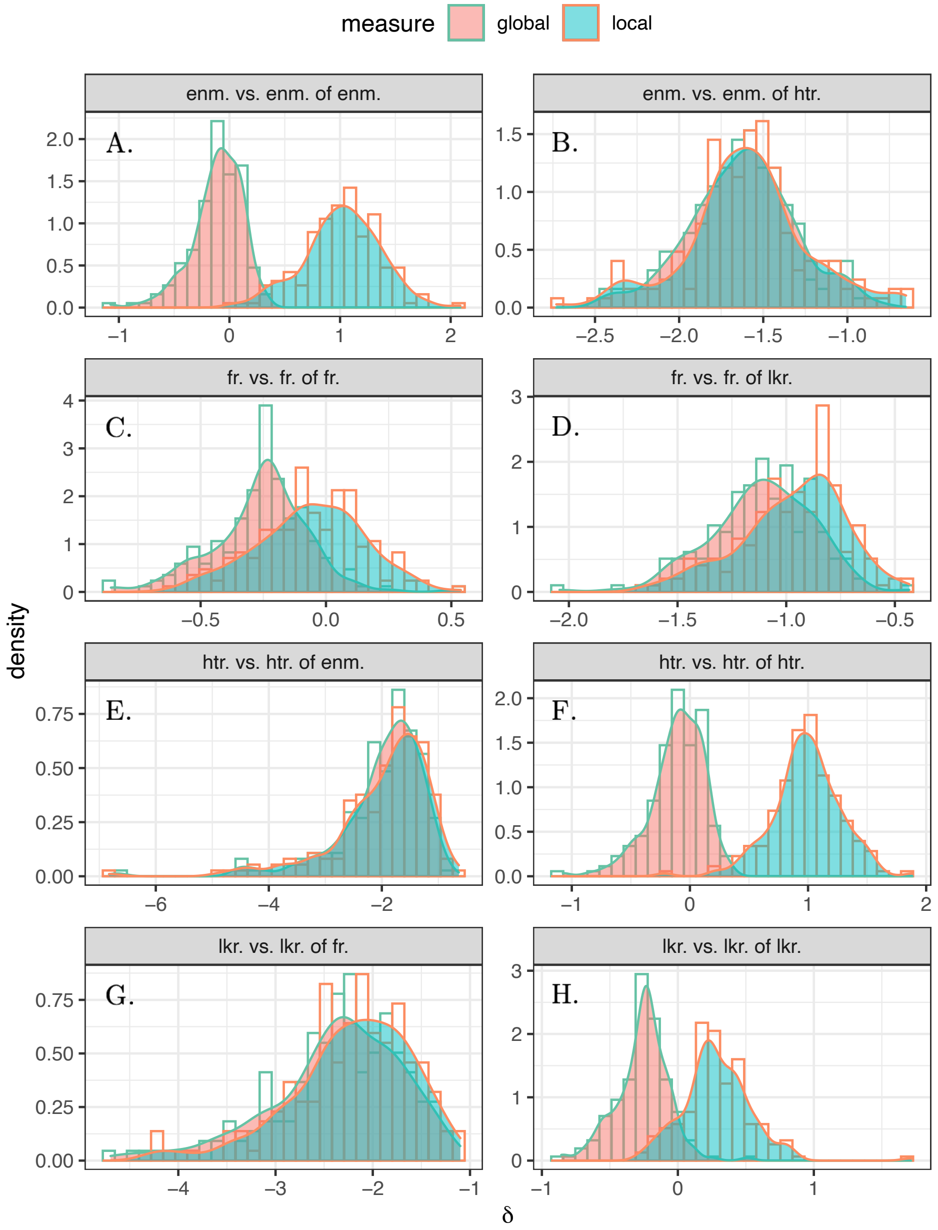

FIG. S4. Histograms of δ_g and δ_l for directed networks among 176 network villages. The difference between the global and local paradoxes can be explained using the generalized inversity measures for directed networks. These (correlations, P-values) for the whole dataset can be summarized as $(-0.03, 4.2e^{-5})$, $(-0.01, 0.2)$, $(-0.06, 2.32e^{-12})$, and $(-0.09, < 2.2e^{-16})$ for the enmity world and $(-0.15, < 2.2e^{-16})$, $(-0.14, < 2.2e^{-16})$, $(-0.19, < 2.2e^{-16})$, and $(-0.16, < 2.2e^{-16})$ for the friendship world. The order of these correlations is for the paradox strengths comparing the number of enemies/friends with the number of enemies/friends of haters/likers (panels B and D); the number of haters/likers with the number of haters/likers of enemies/friends (panels E and G); the number of enemeis/friends with the number of enemies/friends of enemeis/friends (panels A and C); and the number of haters/likers with the number of haters/likers of haters/likers (panels F and H). A detailed analysis of these correlations is provided in Fig. S13.

Section E: Relationship of topological features with paradox strength

Our study examines the relationship of various topological features with friendship and enmity paradox strength using regression modeling. The correlation matrix for both enmity and friendship paradox data frames is shown in Fig. S5. Our regression analysis considers only a subset of these topological features due to their significant correlation, including H_{var}, H_*, $H_{\text{deg-div}}$, H_i, and T_g. The effect of H_a is almost the same as the effect of H_i with a negative sign since they are highly negatively correlated, whereas the effect of T_l is almost the same as that of T_g since they are highly positively correlated. The effect of regression of δ_l and δ_g on different topological features is in Table S3.

	enmity paradox		friendship paradox	
	Model δ_g	Model δ_l	Model δ_g	Model δ_l
H_{var}	-0.76^{***}	-0.66^{***}	-0.88^{***}	-0.88^{***}
	(0.02)	(0.03)	(0.02)	(0.02)
H_*	-0.39^{***}	-0.46^{***}	-0.47^{***}	-0.44^{***}
	(0.03)	(0.04)	(0.02)	(0.02)
$H_{\text{deg-div}}$	0.14^{***}	0.13^{***}	0.12^{***}	0.10^{***}
	(0.02)	(0.02)	(0.03)	(0.03)
H_i	0.16^{***}	-0.05	0.18^{***}	-0.20^{***}
	(0.02)	(0.03)	(0.02)	(0.02)
T_g	0.01	0.03	0.05	0.04
	(0.02)	(0.02)	(0.03)	(0.03)
R^2	0.97	0.94	0.95	0.95
Adj. R^2	0.96	0.93	0.95	0.94
Num. obs.	176	176	176	176

$^{***}p < 0.001$; $^{**}p < 0.01$; $^{*}p < 0.05$

TABLE S3. Regression analysis of δ_g and δ_l of the enmity paradox and δ_g and δ_l of the friendship paradox with respect to different measures. The model considered in our analysis is summarized as $\delta \sim H_{\text{var}} + H_* + H_{\text{deg-div}} + H_i + T_g$.

Figs. S6-S7 illustrate how the global and local enmity and friendship paradox strengths for undirected networks created by reciprocated (ur) and symmetrized edges (us) change with different topological measures. Among the different topological features, H_* and H_{var} have a negative correlation with the strength of the paradoxes. In these figures, H_a shows a positive correlation with strength, and also H_i and $H_{\text{deg-div}}$ show a negative correlation with strength. Among the various measures, H_{var} and H_* are the most significant, with large effects for both undirected (symmetrized) and undirected (reciprocated) networks (see Figs. 6 [main text] and S8). It is expected that the paradoxes become stronger as the variance and starlike embedding increase.

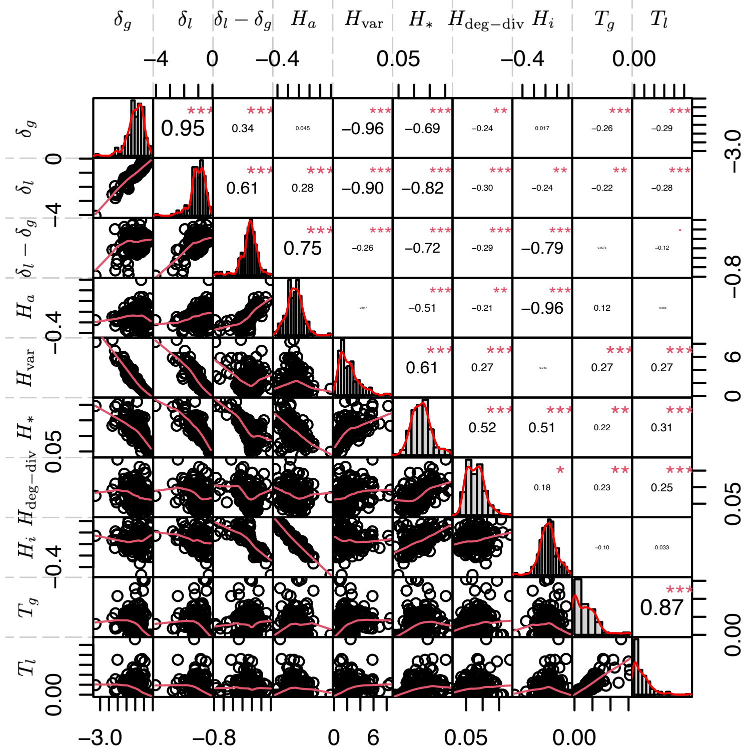

(a) enmity

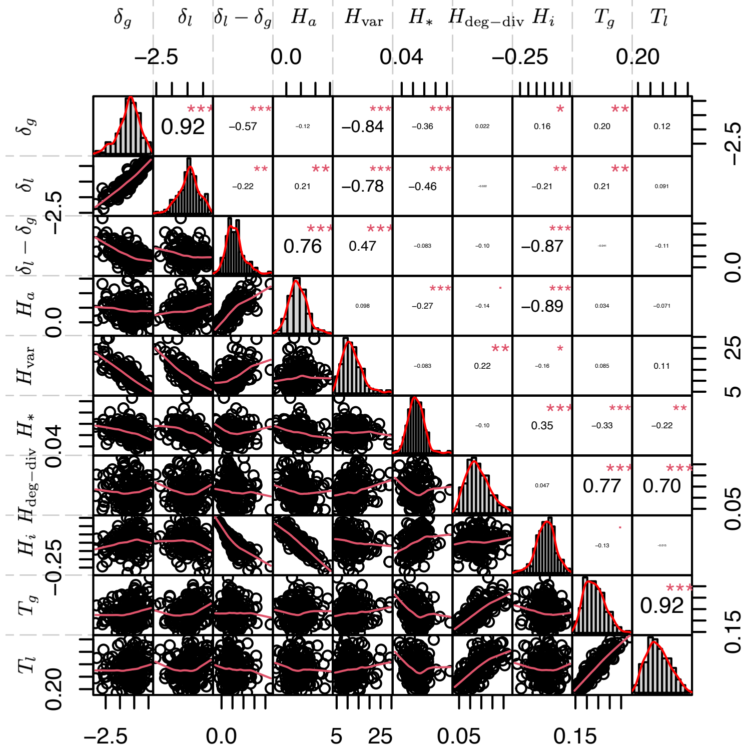

(b) friendship

FIG. S5. Chart of correlation matrices for enmity and friendship data frames.

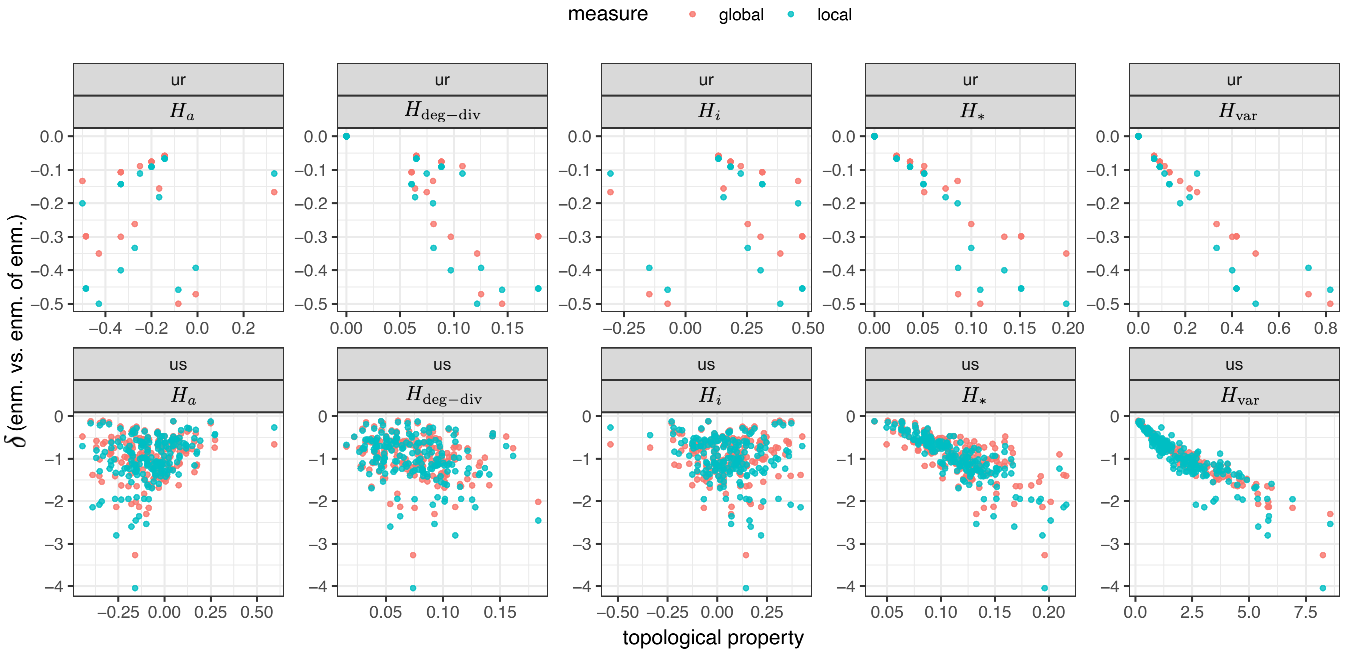

FIG. S6. The strength of the global and local enmity paradox for undirected networks created by reciprocated (ur) and symmetrized edges (us) changes with different topological measures.

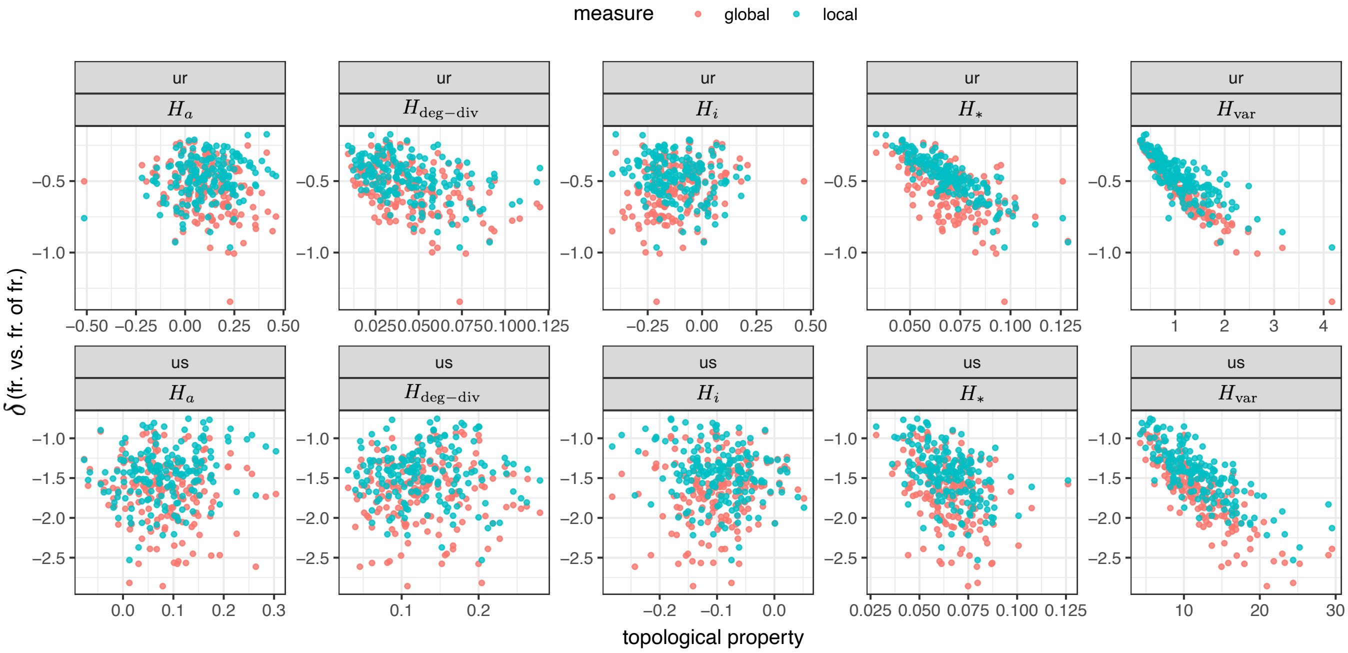

FIG. S7. The strength of the global and local friendship paradox for undirected networks created by reciprocated (ur) and symmetrized edges (us) changes with different topological measures.

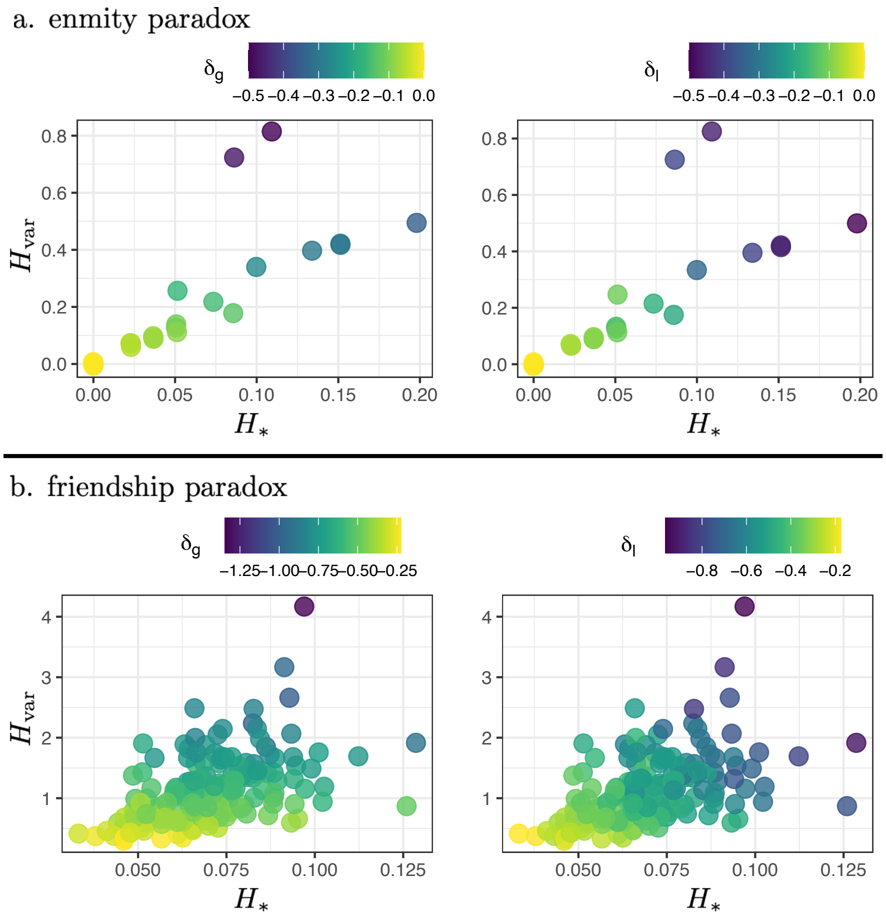

FIG. S8. Critical features in enmity and friendship paradoxes. The heat maps show the global and local paradox strengths for undirected (reciprocated) networks as a function of two measures: degree variance H_{var} and the starlike embedding H_*. The larger the variance and the more the starlike embedding, the stronger the paradox.

Section F: Generalized enmity and friendship paradoxes

Given the vector of non-topological characteristic denoted by x, we define a diagonal matrix D_x with diagonal entries $x_i, i \in \{1, ..., n\}$. Thus, the generalized enmity paradox for the global definition of the difference $\delta_{g,-w}(x)$ can be written as Eq. S25.

$$\delta_{g,-w}(x) = \frac{\mathbf{1}^T D_x \mathbf{1}\mathbf{1}^T A_{(-)}\mathbf{1} - \mathbf{1}^T A_{(-)} D_x \mathbf{1} \cdot \mathbf{1}^T \mathbf{1}}{\mathbf{1}^T A_{(-)}\mathbf{1} \cdot \mathbf{1}^T\mathbf{1}} \tag{S25}$$

The numerator can be reduced to $\mathrm{Tr}\left[D_{(-)}\left(\mathbb{J}D_x\mathbb{J} - D_x\mathbb{J}^2\right)\right] = \mathbf{1}^T D_{(-)}(J - n\mathbb{I})D_x\mathbf{1} = \sum_{i,j} k_{(-),i}x_j - n\sum_i k_{(-),i}\sum_i x_i$, which is negative only if there is a negative correlation between k and x.

The local definition of enmity paradox can also be written as Eq. S26.

$$\begin{aligned}\delta_{l,-w}(x) =& \frac{\mathbf{1}^T D_x \mathbf{1} - \mathbf{1}^T D_{(-)}^{-1} A_{(-)} D_x \mathbf{1}}{\mathbf{1}^T\mathbf{1}} \\ =& \frac{2\mathbf{1}^T D_x\mathbf{1} - \mathbf{1}^T D_{(-)}^{-1}A_{(-)}D_x\mathbf{1} - \mathbf{1}^T D_x A_{(-)} D_{(-)}^{-1}\mathbf{1}}{2\mathbf{1}^T\mathbf{1}}\end{aligned} \tag{S26}$$

Using $\mathbb{I} = D_{(-)}^{-1}D_{(-)}$ and $D_{(-)}\mathbf{1} = A_{(-)}\mathbf{1}$, the numerator can be reduced to

$$\mathrm{Tr}\left[A_{(-)}\left(2\mathbb{J}D_x D_{(-)}^{-1} - D_x\mathbb{J}D_{(-)}^{-1} - D_{(-)}^{-1}\mathbb{J}D_x\right)\right].$$

Here, there are a variety of possibilities; for example, if $x_i < k_i$, this quantity is always negative and all local differences are also negative, then this is merely an uninteresting sufficient condition. The relationship between $\delta_{g,-w}(x)$ and $\delta_{l,-w}(x)$ can be formalized using the edge-based correlation between x_i on one endpoint of edge (i, j) and the inverse degree of another endpoint of that edge, $1/k_{(-),j}$, i.e., $\delta_{g,-w}(x) - \delta_{l,-w}(x) \propto \rho_{(x)}$, where $\rho_{(x)} = \mathrm{cor}(x_i, 1/k_{(-),j} | (i, j) \in E_{(-)})$ [12]. If the aforementioned edge-based correlation is positive, we have $\delta_{l,-w}(x) < \delta_{g,-w}(x)$; if it is negative, we have $\delta_{g,-w}(x) < \delta_{l,-w}(x)$; and if there is no correlation, then two measures are equivalent. Using this relationship we have four possibilities of (positive, positive), (positive, negative), (negative, positive), and (negative, negative) for $(\delta_{l,-w}(x), \delta_{g,-w}(x))$ differences.

The results for global and local measures of the generalized enmity and friendship paradoxes for undirected (symmetrized) networks are provided in Tables S4 and S5. The distribution of these measures over 176 village networks in the Honduras dataset is represented in Fig. S9. The difference between the global and local measures can be explained through the inversity measure defined as the following correlation: $\rho_{-w/+w,(x)} = \mathrm{cor}(x_i, 1/k_{(-/+),j} | (i, j) \in E_{(-/+)})$, i.e., $\delta_{g,-w/+w}(x) - \delta_{l,-w/+w}(x) \propto \rho_{-w/+w,(x)}$. The distribution of these inversity measures and the P-values for 176 village networks in the Honduras dataset is provided in Fig. S10. The results show the generalized paradoxes in both enmity and friendship paradoxes (e.g., wealth). Due to the higher correlation between topological features and positive degrees, generalized friendship paradoxes are stronger than generalized enmity paradoxes. We see an agreement in the existence of the generalized paradoxes for different attributes between the enmity and friendship networks. A further investigation of the generalized enmity paradox is left for future study.

attribute	random ppl. in enmity network (n = 2857)	enmity: enemy of of random ppl. (n = 2816)	enmity: enemy of enemy of random ppl. (n = 2816)	hypothesis: $\delta_x < 0$ estimate (significant) (1st order)	(2nd order)
**(global)**					
age	33.33 ± 2.81	34.19 ± 3.34	34.41 ± 3.62	-0.86 (****)	-1.08 (****)
wealth (household)	2.88 ± 0.86	2.94 ± 0.88	2.95 ± 0.88	-0.06 (****)	-0.06 (****)
health	2.68 ± 0.23	2.62 ± 0.26	2.62 ± 0.27	0.05	0.05
mental health	2.76 ± 0.25	2.72 ± 0.29	2.72 ± 0.29	0.04	0.04
no little interest	11.35 ± 0.80	11.21 ± 0.94	11.22 ± 0.95	0.14	0.13
no feeling down	11.58 ± 0.71	11.40 ± 0.85	11.38 ± 0.86	0.19	0.21
**(local)**					
age	33.33 ± 2.81	33.85 ± 3.32	33.71 ± 2.95	-0.52 (****)	-0.38 (****)
wealth (household)	2.88 ± 0.86	2.94 ± 0.87	2.92 ± 0.87	-0.06 (****)	-0.04 (****)
health	2.68 ± 0.23	2.62 ± 0.26	2.65 ± 0.24	0.05	0.02
mental health	2.76 ± 0.25	2.72 ± 0.30	2.74 ± 0.26	0.04	0.02
no little interest	11.34 ± 0.80	11.21 ± 1.00	11.29 ± 0.85	0.14	0.06
no feeling down	11.58 ± 0.71	11.43 ± 0.86	11.51 ± 0.74	0.16	0.07

$^{(****)}p < 0.001$; $^{(***)}p < 0.001$; $^{(**)}p < 0.01$; $^{(*)}p < 0.05$

TABLE S4. The empirical validation for global and local generalized enmity paradox for undirected (symmetrized) networks. The average of demographic qualities of random people in comparison with the average of demographics for neighbors of random people and neighbors' neighbors of random people. The "no little interest" and "no feeling down" are measured over the most recent 2 weeks.

attribute	random ppl. in friendship network (n = 2821)	friendship: friend of of random ppl. (n = 2816)	friendship: friend of friend of random ppl. (n = 2816)	hypothesis: $\delta_x < 0$ estimate (significant) (1st order)	(2nd order)
**(global)**					
age	32.60 ± 2.25	34.70 ± 2.67	35.30 ± 2.95	-2.1 (****)	-2.7 (****)
wealth (household)	2.85 ± 0.85	2.98 ± 0.89	2.98 ± 0.89	-0.13 (****)	-0.13 (****)
health	2.73 ± 0.19	2.69 ± 0.21	2.68 ± 0.21	0.03	0.04
mental health	2.84 ± 0.21	2.81 ± 0.23	2.81 ± 0.24	0.03	0.03
no little interest	11.56 ± 0.72	11.49 ± 0.74	11.48 ± 0.76	0.06	0.07
no feeling down	11.82 ± 0.59	11.73 ± 0.67	11.72 ± 0.68	0.09	0.1
**(local)**					
age	32.60 ± 2.25	34.29 ± 2.42	34.64 ± 2.58	-1.7 (****)	-2.04 (****)
wealth (household)	2.85 ± 0.85	2.97 ± 0.88	2.96 ± 0.88	-0.12 (****)	-0.11 (****)
health	2.73 ± 0.19	2.70 ± 0.21	2.69 ± 0.21	0.03	0.03
mental health	2.84 ± 0.21	2.82 ± 0.23	2.81 ± 0.23	0.02	0.03
no little interest	11.56 ± 0.72	11.50 ± 0.74	11.50 ± 0.74	0.06	0.06
no feeling down	11.82 ± 0.59	11.73 ± 0.66	11.74 ± 0.65	0.09	0.08

$^{(****)}p < 0.001$; $^{(***)}p < 0.001$; $^{(**)}p < 0.01$; $^{(*)}p < 0.05$

TABLE S5. The empirical validation for global and local generalized friendship paradox for undirected (symmetrized) networks. The average of demographic qualities of random people in comparison with the average of demographics for neighbors of random people and neighbors' neighbors of random people. The "no little interest" and "no feeling down" are measured over the most recent 2 weeks.

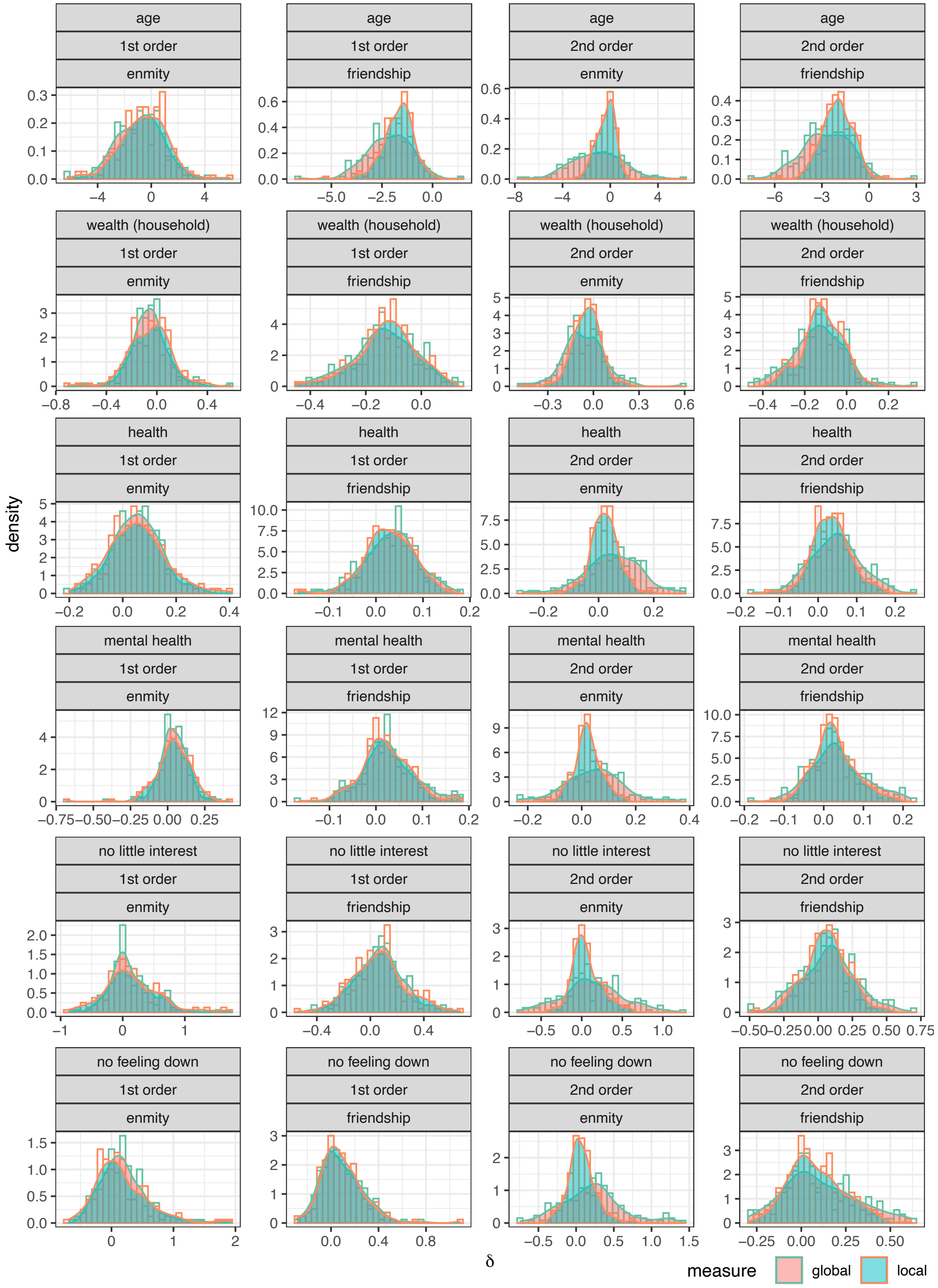

FIG. S9. Histograms of global and local measures, δ_g and δ_l, for the generalized enmity and friendship paradoxes in undirected (symmetrized) networks. Here, we provide these measures for both the first and second order neighbors.

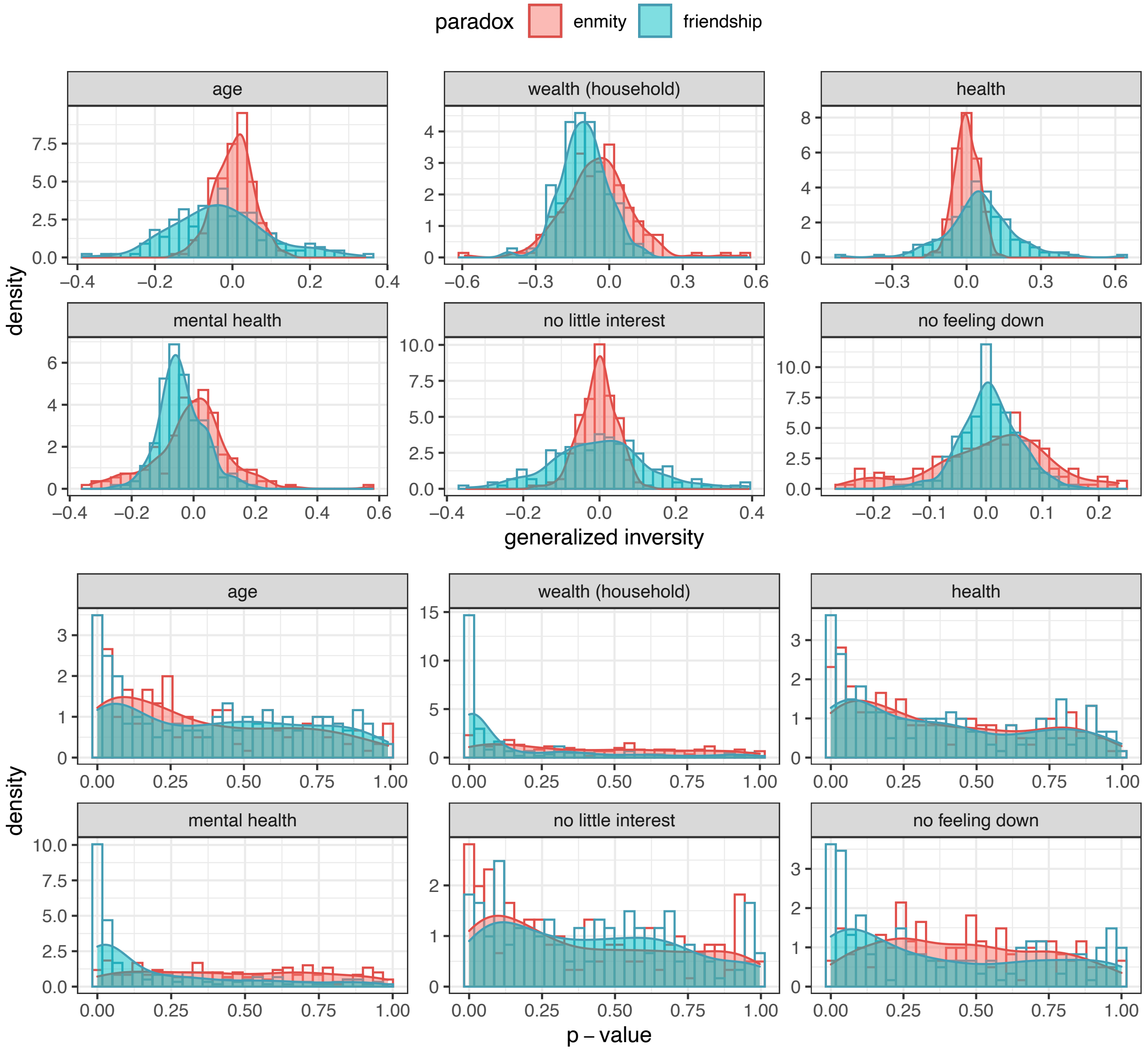

FIG. S10. The village-level inversity distribution for undirected (symmetrized) networks. These inversities are aligned with the inversity measures defined in order to explain the differences between global and local measures of generalized enmity and friendship paradoxes. The distribution of these correlations over 176 village networks in the Honduras dataset is represented in the two upper rows, while the P-values of these correlation tests are shown in the two bottom rows.

Section G: Generalized inversity

To find the relationships between the global and local paradox strengths in the mixed worlds, i.e., $\delta_{g,-w}(+)-\delta_{l,-w}(+)$ and $\delta_{g,+w}(-)-\delta_{l,+w}(-)$, we generalize the inversity measure originally proposed for a friendship network in Ref. [5]. The difference between $\delta_{g,-w}(+)$ and $\delta_{l,-w}(+)$ can be written as Eq. S27.

$$\delta_{g,-w}(+)-\delta_{l,-w}(+)=\frac{\mathbf{1}^T D_{(-)}^{-1} A_{(-)} D_{(+)} \mathbf{1}\cdot\mathbf{1}^T A_{(-)}\mathbf{1}-\mathbf{1}^T A_{(+)} A_{(-)}\mathbf{1}\cdot\mathbf{1}^T\mathbf{1}}{\mathbf{1}^T A_{(-)}\mathbf{1}\cdot\mathbf{1}^T\mathbf{1}} \tag{S27}$$

$$=\frac{\sum_{(i,j)\in E_{(-)}} {}^{k_{(+),j}}/_{k_{(-),i}}}{n}-\frac{\sum_i k_{(+),i}k_{(-),i}}{2|E_{(-)}|} \tag{S28}$$

The generalized inversity for the mixed world of $-w$ is defined as the correlation between the positive degree of node i and the inverse negative degree of node j on the two endpoints of a random negative edge $(i,j)\in E_{(-)}$, which is derivable as follows: Using a similar convention as in Ref. [5], we denote the positive degree variable corresponding to one endpoint as $k_{D,(+)}$ and the inverse negative degree of the other endpoint as $k_{ID,(-)}$. The generalized inversity in this mixed world can be written as the following equation,

$$\begin{aligned}
\rho_{\text{mixed,D(+),ID(}-)} &= \frac{1}{2|E_{(-)}|\sigma_{D,(+)}\sigma_{ID,(-)}}\sum_{(i,j)\in E_{(-)}}\left(k_{(+),i}-\mu_{D,(+)}\right)\left({}^1/_{k_{(-),j}}-\mu_{ID,(-)}\right)\\
&= \frac{1}{2|E_{(-)}|\sigma_{D,(+)}\sigma_{ID,(-)}}\sum_{(i,j)\in E_{(-)}}\left({}^{k_{(+),i}}/_{k_{(-),j}}-k_{(+),i}\mu_{ID,(-)}-{}^{\mu_{D,(+)}}/_{k_{(-),j}}+\mu_{D,(+)}\mu_{ID,(-)}\right)\\
&= \frac{1}{2|E_{(-)}|\sigma_{D,(+)}\sigma_{ID,(-)}}\sum_{(i,j)\in E_{(-)}}\left({}^{k_{(+),i}}/_{k_{(-),j}}-k_{(+),i}\mu_{ID,(-)}\right)\\
&= \frac{n}{2|E_{(-)}|\sigma_{D,(+)}\sigma_{ID,(-)}}\left(\frac{\sum_{(i,j)\in E_{(-)}} {}^{k_{(+),i}}/_{k_{(-),j}}}{n}-\frac{\sum_i k_{(+),i}k_{(-),i}}{2|E_{(-)}|}\right),
\end{aligned} \tag{S29}$$

where the first two moments of $k_{D,(+)}$ and $k_{ID,(-)}$ in Eq. S29 are computed as follows:

$$\mu_{D,(+)}=\frac{\sum_{(i,j)\in E_{(-)}} k_{(+),i}}{2|E_{(-)}|}=\frac{\sum_i k_{(+),i}k_{(-),i}}{2|E_{(-)}|} \tag{S30}$$

$$\mu_{ID,(-)}=\frac{\sum_{(i,j)\in E_{(-)}} {}^1/_{k_{(-),j}}}{2|E_{(-)}|}=\frac{n}{2|E_{(-)}|} \tag{S31}$$

$$\sigma_{D,(+)}=\frac{\sum_{(i,j)\in E_{(-)}}(k_{(+),i}-\mu_{D,(+)})^2}{2|E_{(-)}|}=\frac{\sum_i(k_{(+),i}-\mu_{D,(+)})^2 k_{(-),i}}{2|E_{(-)}|} \tag{S32}$$

$$\sigma_{ID,(-)}=\frac{\sum_{(i,j)\in E_{(-)}}({}^1/_{k_{(-),j}}-\mu_{ID,(-)})^2}{2|E_{(-)}|}=\frac{\sum_{j:k_{(-),j}>0} {}^1/_{k_{(-),j}}-2n\mu_{ID,(-)}+2|E_{(-)}|\mu_{ID,(-)}^2}{2|E_{(-)}|}. \tag{S33}$$

where, in Eq. S29 and other equations regarding the generalized inversity for the mixed world of $-w$, n denotes the size of nodes with non-zero negative degrees. For the mixed world of $+w$, n denotes the size of nodes with non-zero positive degrees.

Therefore, Eq. S29 can be written as Eq. S34,

$$\delta_{g,-w}(+)-\delta_{l,-w}(+)=\rho_{\text{mixed,D(+),ID(}-)}\sigma_{D,(+)}\sigma_{ID,(-)}\overline{k_{(-)}}, \tag{S34}$$

where, $\overline{k_{(-)}}={}^{2|E_{(-)}|}/_n$ and $\rho_{\text{mixed,D(+),ID(}-)}=cor(k_{(+),i},{}^1/_{k_{(-),j}}|(i,j)\in E_{(-)})$. Similarly, the difference $\delta_{l,+w}(-)$ and $\delta_{g,+w}(-)$ can be written as Eq. S35.

$$\delta_{g,+w}(-)-\delta_{l,+w}(-)=\frac{\mathbf{1}^T D_{(+)}^{-1} A_{(+)} D_{(-)} \mathbf{1}\cdot\mathbf{1}^T A_{(+)}\mathbf{1}-\mathbf{1}^T A_{(+)} A_{(-)}\mathbf{1}\cdot\mathbf{1}^T\mathbf{1}}{\mathbf{1}^T A_{(+)}\mathbf{1}\cdot\mathbf{1}^T\mathbf{1}} \tag{S35}$$

$$=\frac{\sum_{(i,j)\in E_{(+)}} {}^{k_{(-),j}}/_{k_{(+),i}}}{n}-\frac{\sum_i k_{(+),i}k_{(-),i}}{2|E_{(+)}|} \tag{S36}$$

And, through similar derivation, it can be shown that

$$\delta_{g,+w}(-) - \delta_{l,+w}(-) = \rho_{\text{mixed,D}(-),\text{ID}(+)}\sigma_{D,(-)}\sigma_{ID,(+)}\overline{k_{(+)}}\,, \tag{S37}$$

where, $\overline{k_{(+)}} = {}^{2|E_{(+)}|}/_{n}$ and $\rho_{\text{mixed,D}(-),\text{ID}(+)} = cor(k_{(-),i}, {}^{1}/_{k_{(+),j}}|(i,j) \in E_{(+)})$.

For directed networks, we can similarly derive the equations corresponding to the inversity measure as follows. We only present the proof for the difference between $\delta_{g,in-w}(out)$ and $\delta_{l,in-w}(out)$.

$$\begin{aligned}\delta_{g,in-w}(out) - \delta_{l,in-w}(out) &= \frac{\mathbf{1}^T D_{\text{in}}^{-1} A^T D_{\text{out}}\mathbf{1}\cdot\mathbf{1}^T A\mathbf{1} - \mathbf{1}^T A^T A\mathbf{1}\cdot\mathbf{1}^T\mathbf{1}}{\mathbf{1}^T A\mathbf{1}\cdot\mathbf{1}^T\mathbf{1}} &\text{(S38)}\\ &= \frac{\sum_{(i,j)\in E} {}^{k_{i,out}}/_{k_{j,in}}}{n} - \frac{\sum_i k_{i,out}^2}{|E|} &\text{(S39)}\end{aligned}$$

The inversity in this scenario is defined as the correlation between the out-degree of node i and the inverse in-degree of node j on the two endpoints of a random edge $(i,j) \in E$, which can be derived as follows. We denote the out-degree variable corresponding to one endpoint as $k_{D,out}$ and the inverse in-degree of the other endpoint as $k_{ID,in}$. The inversity measure can be expanded for this directed network via the following equation,

$$\begin{aligned}\rho_{\text{mixed,D(out),ID(in)}} &= \frac{1}{|E|\sigma_{D,(out)}\sigma_{ID,(in)}}\sum_{(i,j)\in E}\left(k_{i,out} - \mu_{D,(out)}\right)\left({}^{1}/_{k_{j,in}} - \mu_{ID,(in)}\right)\\ &= \frac{1}{|E|\sigma_{D,(out)}\sigma_{ID,(in)}}\sum_{(i,j)\in E}\left({}^{k_{i,out}}/_{k_{j,in}} - k_{i,out}\mu_{ID,(in)} - {}^{\mu_{D,(out)}}/_{k_{j,in}} + \mu_{D,(out)}\mu_{ID,(in)}\right)\\ &= \frac{1}{|E|\sigma_{D,(out)}\sigma_{ID,(in)}}\sum_{(i,j)\in E}\left({}^{k_{i,out}}/_{k_{j,in}} - k_{i,out}\mu_{ID,(in)}\right)\\ &= \frac{n}{|E|\sigma_{D,(out)}\sigma_{ID,(in)}}\left(\frac{\sum_{(i,j)\in E} {}^{k_{i,out}}/_{k_{j,in}}}{n} - \frac{\sum_{i:k_{i,in}>0} k_{i,out}k_{i,in}}{|E|}\right)\,, &\text{(S40)}\end{aligned}$$

where the first two moments of $k_{D,(out)}$ and $k_{ID,(in)}$ in Eq. S40 are computed as follows:

$$\begin{aligned}\mu_{D,(out)} &= \frac{\sum_{(i,j)\in E} k_{i,out}}{|E|} = \frac{\sum_i k_{i,out}^2}{|E|} &\text{(S41)}\\ \mu_{ID,(in)} &= \frac{\sum_{(i,j)\in E} {}^{1}/_{k_{j,in}}}{|E|} = \frac{n}{|E|} &\text{(S42)}\\ \sigma_{D,(out)} &= \frac{\sum_{(i,j)\in E}(k_{i,out} - \mu_{D,(out)})^2}{|E|} = \frac{\sum_i (k_{i,out} - \mu_{D,(out)})^2 k_{i,out}}{|E|} &\text{(S43)}\\ \sigma_{ID,(in)} &= \frac{\sum_{(i,j)\in E}({}^{1}/_{k_{j,in}} - \mu_{ID,(in)})^2}{|E|} = \frac{\sum_{j:k_{j,in}>0} {}^{1}/_{k_{j,in}} - 2n\mu_{ID,(in)} + 2|E|\mu_{ID,(in)}^2}{|E|}\,. &\text{(S44)}\end{aligned}$$

$$\delta_{g,in-w}(out) - \delta_{l,in-w}(out) = \rho_{\text{mixed,D(out),ID(in)}}\sigma_{D,(out)}\sigma_{ID,(in)}\overline{k}\,, \tag{S45}$$

where, $\overline{k} = {}^{2|E|}/_{n}$ and $\rho_{\text{mixed,D(out),ID(in)}} = cor(k_{i,out}, {}^{1}/_{k_{j,in}}|(i,j) \in E)$.

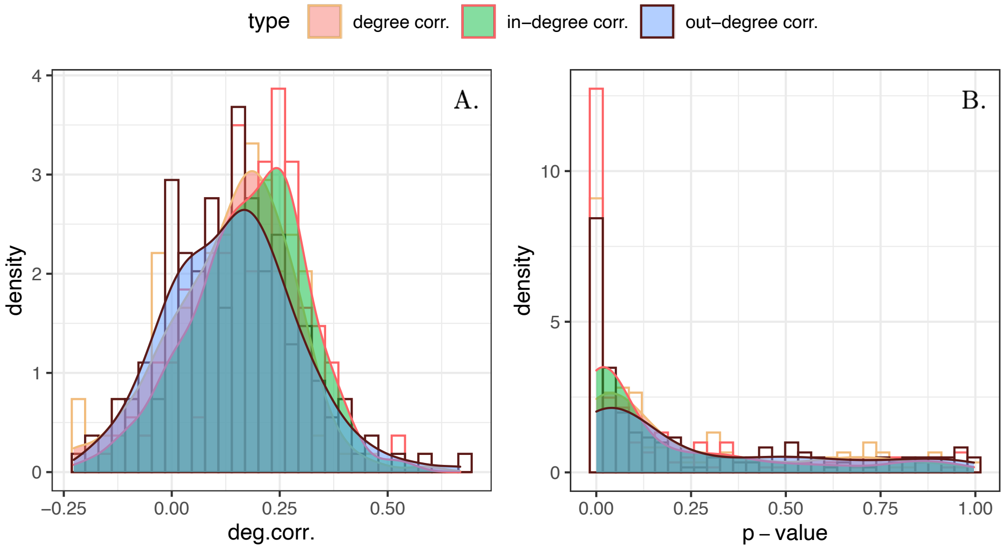

FIG. S11. Correlation between positive and negative degree. The distribution of correlation between positive and negative degrees over 176 village networks in the Honduras dataset is represented in panel A, while the P-values of these correlation tests for these networks are represented in panel B.

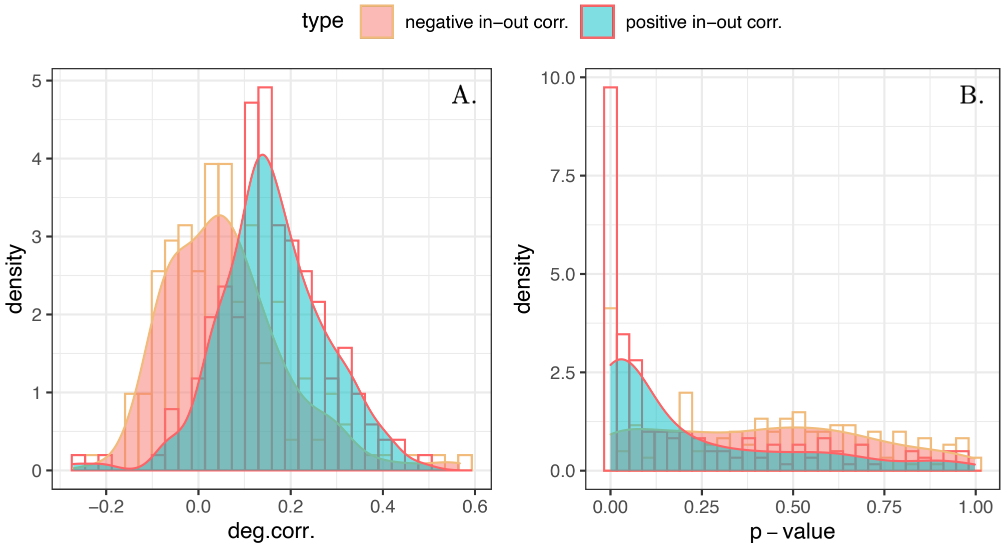

FIG. S12. Correlation between in- and out-degrees for positive and negative networks. The distribution of correlation between in- and out-degrees over 176 enmity and friendship village networks in the Honduras dataset is represented in panel A, while the P-values of these correlation tests for these networks are represented in panel B.

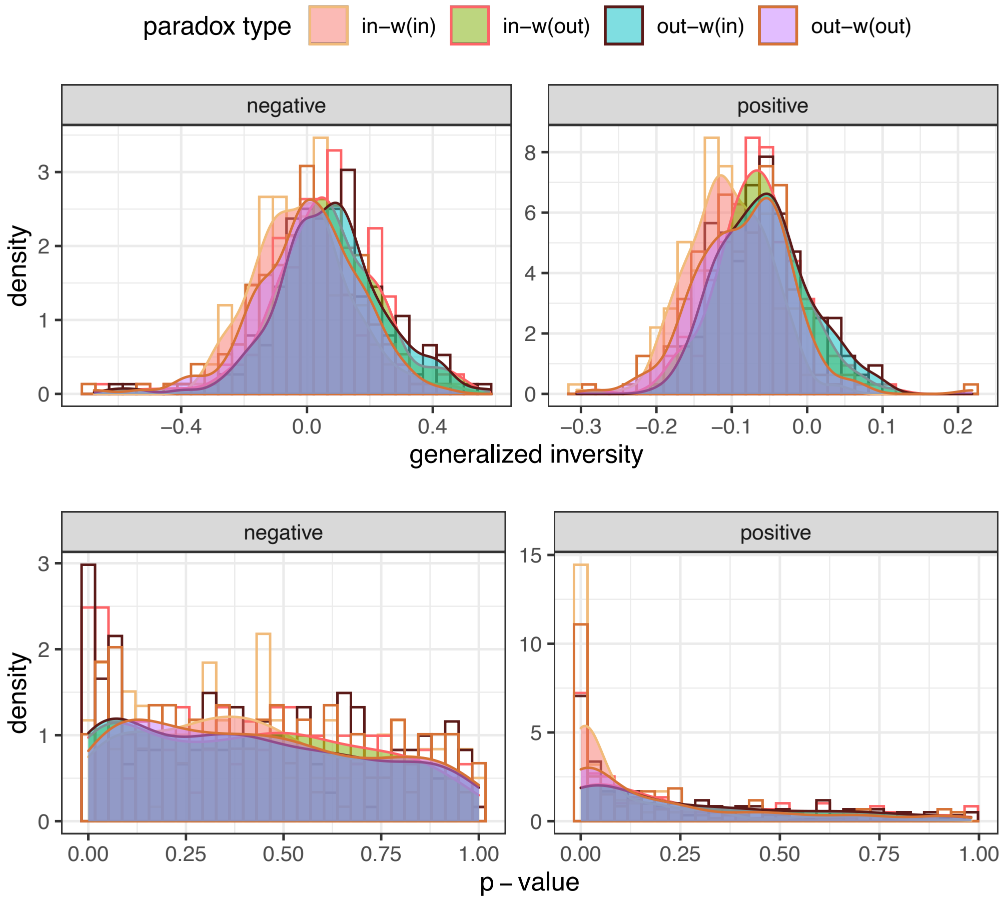

FIG. S13. The village-level inversities associated with four cases of enmity and friendship paradoxes in directed networks. These inversities are aligned with the four cases introduced in Section D, i.e., $cor(k_{i,out}, 1/k_{j,in}|(i,j) \in E)$, $cor(1/k_{i,out}, k_{j,in}|(i,j) \in E)$, $cor(1/k_{i,out}, k_{j,out}|(i,j) \in E)$, and $cor(k_{i,in}, 1/k_{j,in}|(i,j) \in E)$ in order. The distribution of these correlations over 176 enmity and friendship village networks in the Honduras dataset is represented in the upper row, while the P-values of these correlation tests for these networks are represented in the bottom row. These (correlations, P-values) for the whole dataset can be summarized as $(-0.03, 4.2e^{-5})$, $(-0.01, 0.2)$, $(-0.06, 2.32e^{-12})$, and $(-0.09, < 2.2e^{-16})$ for enmity world and $(-0.15, < 2.2e^{-16})$, $(-0.14, < 2.2e^{-16})$, $(-0.19, < 2.2e^{-16})$, and $(-0.16, < 2.2e^{-16})$ for friendship world.

[1] H. B. Shakya, D. Stafford, D. A. Hughes, T. Keegan, R. Negron, J. Broome, M. McKnight, L. Nicoll, J. Nelson, E. Iriarte, *et al.*, Exploiting social influence to magnify population-level behaviour change in maternal and child health: study protocol for a randomised controlled trial of network targeting algorithms in rural honduras, BMJ open **7**, e012996 (2017).

[2] R. Tourangeau, L. J. Rips, and K. Rasinski, *The psychology of survey response* (Cambridge University Press, 2000).

[3] P. J. Shoemaker, M. Eichholz, and E. A. Skewes, Item nonresponse: Distinguishing between don't know and refuse, International Journal of Public Opinion Research **14**, 193 (2002).

[4] N. Harrigan and J. Yap, Avoidance in negative ties: Inhibiting closure, reciprocity, and homophily, Social Networks **48**, 126 (2017).

[5] V. Kumar, D. Krackhardt, and S. Feld, Interventions with inversity in unknown networks can help regulate contagion, preprint arXiv:2105.08758 (2021).

[6] G. T. Cantwell, A. Kirkley, and M. E. J. Newman, The friendship paradox in real and model networks, Journal of Complex Networks **9**, cnab011 (2021).

[7] M. E. J. Newman, Mixing patterns in networks, Physical review E **67**, 026126 (2003).

[8] E. Estrada, Quantifying network heterogeneity, Physical Review E **82**, 066102 (2010).

[9] R. Jacob, K. Harikrishnan, R. Misra, and G. Ambika, Measure for degree heterogeneity in complex networks and its application to recurrence network analysis, Royal Society open science **4**, 160757 (2017).

[10] V. Latora, V. Nicosia, and G. Russo, *Complex networks: principles, methods and applications* (Cambridge University Press, 2017).

[11] L. C. Freeman, Centrality in social networks: Conceptual clarification, Social network: critical concepts in sociology. Londres: Routledge **1**, 238 (2002).

[12] N. Alipourfard, B. Nettasinghe, A. Abeliuk, V. Krishnamurthy, and K. Lerman, Friendship paradox biases perceptions in directed networks, Nature communications **11**, 1 (2020).
